# Distinct roles of histone H2B ubiquitination at promoters and coding regions of Pol II-transcribed stress genes

**DOI:** 10.1186/s13059-025-03891-1

**Published:** 2025-12-09

**Authors:** Rubén Barrios, Montserrat Vega, Rebeca Gracia-Domingo, Susanna Boronat, Sarela García-Santamarina, Jason C. Tanny, José Ayté, Elena Hidalgo

**Affiliations:** 1 Oxidative Stress and Cell Cycle Group, Universitat Pompeu Fabra, C/Dr. Aiguader 88, Barcelona, 08003 Spain; 2 Department of Pharmacology and Therapeutics, McGill University, Montreal, Canada; 3 Present address: Leibniz Institute for Natural Product Research and Infection Biology - Hans-Knöll-Institute, Beutenbergstraße 11A, Jena, 07745 Germany

**Keywords:** H2B ubiquitination, H2B deubiquitinases, Stress genes, Nucleosome dynamics, *S. pombe*

## Abstract

**Background:**

The histone code, comprised of diverse histone post-translational modifications, intricately regulates nucleosome organization and gene expression. Histone marks also serve as binding sites for a diverse array of protein complexes and enzymes, orchestrating downstream cellular functions. Some modifications are crucial for enabling the complete activation of alternative gene expression programs in response to environmental changes.

**Results:**

Our study delves into the consequences of depleting histone mark erasers, the H2B deubiquitinases Ubp8 and Ubp16, in transcription efficiency in fission yeast. Cells lacking these enzymes exhibit resistance to oxidative stress, attributed to enhanced transcription of stress genes both under basal and induced conditions. Ubp8 is recruited to stress promoters as part of the activating SAGA complex, which also acetylates histone H3, while Ubp16 promotes H2B deubiquitination in coding regions. Cells lacking Ubp8 or Ubp16 display enhanced levels of both H2Bub and H3K4me, and chromatin structure at stress genes is already affected under basal conditions and is not timely restored in the wake of elongating Pol II. Depletion of the COMPASS complex, required to methylate H3K4, solely abolishes stress resistance in Δ*ubp16*. In contrast, cells lacking Ubp8 display independence from SAGA or COMPASS for full transcriptional activation, suggesting an essential and sufficient role of H2Bub in Pol II promoter escape.

**Conclusions:**

This study provides insights into the complex histone crosstalk regulating gene expression and underscores the pivotal role of histone ubiquitination in promoting efficient nucleosome dynamics during Pol II transcription.

**Supplementary Information:**

The online version contains supplementary material available at 10.1186/s13059-025-03891-1.

## Background

Gene transcription is tightly regulated in eukaryotes, with chromatin playing a key role in controlling gene expression. The nucleosome, composed of an octamer of histones (H2A, H2B, H3, and H4), serves as the fundamental unit of chromatin structure [1]. DNA wraps around nucleosomes, limiting transcription [2], while histone post-translational modifications (PTMs) modulate chromatin accessibility. These modifications, including ubiquitination, methylation, and acetylation, influence nucleosome architecture and transcription dynamics by recruiting or repelling transcription factors and chromatin remodelers [3]. Histone PTMs function through "writers", "readers" and "erasers" — protein complexes that introduce, interpret, and remove these marks, respectively. Additionally, histone crosstalk, or the interplay between different PTMs, further refines gene regulation.

RNA polymerase II (Pol II)-mediated transcription is closely linked to histone PTMs, which are conserved across genes and species. Pol II transcription rates and the phosphorylation of its C-terminal domain (particularly at serine 5 and 2) correlate with certain histone PTMs [4]. During transcription initiation, specific histone modifications localize to defined regions. For instance, active genes exhibit peaks of histone H3 lysine 4 trimethylation (H3K4me3) and histone H3 acetylation (H3K9,K14ac) at transcription start sites (TSS). Monoubiquitination of histone H2B (H2Bub), one of the largest histone PTMs, spans from gene promoters to termination sites [5] and plays a key role in transcription elongation. While histone-modifying enzymes introduce or remove these marks to regulate transcription, the effects of their absence remain incompletely understood.

H2B ubiquitination is an important mark in transcription regulation, particularly during elongation. It is closely associated with elongating Pol II and facilitates the methylation of histone H3 at lysines 4 and 79 by COMPASS and Dot1, respectively [6]. This suggests that H2Bub enhances transcription elongation by promoting the deposition of additional histone marks associated with actively transcribed genes. However, paradoxically, the absence of H2B ubiquitination has been linked to transcriptional derepression in certain contexts [7], as well as increased antisense transcription in fission yeast [8, 9]. This dual role raises questions about how H2Bub fine-tunes gene expression.

H2Bub is deposited by conserved E3 ligase complexes. In *Schizosaccharomyces pomb*e, the H2B ubiquitin ligase complex (HULC), which consists of Brl1, Brl2, Rhp6, and Shf1, catalyzes H2B ubiquitination at lysine 119 (K119) [10, 11]. HULC recruitment to promoters requires the transcription kinase P-TEFb/Cdk9, which also phosphorylates the Pol II C-terminal domain and the transcription factor Spt5 [12]. Through this mechanism, Cdk9 helps recruit the PAF complex, likely via Spt5 phosphorylation. Rtf1, a PAF subunit, directly interacts with Spt5-P and has a strong functional connection with HULC (for reviews, see [5, 13]). Beyond histones, HULC also ubiquitinates PCNA, highlighting its broader regulatory role [14, 15].

Although not extensively studied in higher eukaryotes [16–18], H2Bub deubiquitination has been widely analyzed in *Saccharomyces cerevisiae*. After being introduced during transcription initiation and elongation, it is rapidly removed by deubiquitinases (DUBs) such as Ubp8, a subunit of the Spt-Ada-Gcn5 acetyltransferase (SAGA) complex [19]. The SAGA DUB module, comprising Ubp8, Sgf11, Sus1, and Sgf73, has a resolved structure and plays distinct genomic roles [19, 20]. Another budding yeast DUB, Ubp10, is involved in gene silencing at telomeres and rDNA [21], distinguishing it from Ubp8, which primarily regulates euchromatic transcription. While both DUBs contribute to the global balance of H2Bub [22], their deletion affects distinct genomic targets [23].

H2Bub interacts with multiple transcription-related complexes (for a review, see [13]), including SAGA, which has dual roles in histone acetylation (via Gcn5) and deubiquitination (via Ubp8). In *S. pombe*, Gcn5 and Mst2 introduce the H3K9ac mark at expressed genes [24–26]. The co-occurrence of histone acetylation and deubiquitination within SAGA suggests a functional interplay, though whether SAGA directly "reads" H2Bub remains unclear.

H2Bub also facilitates the recruitment of histone-modifying enzymes such as Set1-COMPASS, Dot1, and Set2, which methylate H3K4, H3K79, and H3K36, respectively. Set1-COMPASS, an eight-subunit complex, establishes the H3K4 methylation mark, enriched at TSS and dependent on prior H2Bub deposition (for a review, see [13]). While H3K4me typically marks active genes, it exhibits repressive functions in certain chromosomal regions, such as *ste11* and heterochromatin foci in *S. pombe* [7, 27, 28]. Notably, loss of H2Bub has stronger effects on gene regulation than loss of Set1-dependent H3K4me [12].

Beyond transcription, H2Bub influences chromatin structure. The Facilitates Chromatin Transcription (FACT) complex, composed of Spt16 and Pob3 in *S. pombe*, is a putative reader of H2Bub. FACT maintains nucleosome integrity during transcription by mediating the eviction and redeposition of H2A/H2B dimers [13, 29]. In budding yeast, H2Bub deubiquitination by Ubp10 aids FACT in reassembling nucleosomes in the wake of Pol II [30]. Supporting this, yeast mutants expressing a non-ubiquitinatable H2B (H2BK123A) exhibit defective nucleosome reassembly, leading to reduced nucleosome occupancy [31]. In vitro studies further show that the SAGA DUB module deubiquitinates H2B within nucleosomes and in FACT-associated H2A/H2B dimers, suggesting that H2Bub deubiquitination operates at multiple stages of nucleosome disassembly and reassembly [19].

H2Bub plays key roles in gene regulation, yet its precise functions remain unresolved. To further explore its impact, we used oxidative stress tolerance as a readout of Pol II efficiency: switching transcription of stress genes from off to on is required for cellular adaptation to hydrogen peroxide (H_2_O_2_). We examined the transcriptional response of stress genes in *S. pombe* strains lacking the DUBs Ubp8 (a SAGA subunit) or Ubp16 (ortholog of Ubp10 of budding yeast). These mutants displayed increased resistance to peroxide stress, with higher basal expression of stress genes. RNA sequencing revealed that stress genes were more strongly induced in ∆*ubp8* and ∆*ubp16* mutants than in wild-type cells. Both strains exhibited consistently high levels of H2Bub and H3K4me. Ubp8 was recruited to stress gene promoters along with SAGA, while Ubp16 associated with gene bodies after stress induction. Deleting the genes coding for the histone modifiers Gcn5 or Set1 suppressed the stress resistance phenotype of ∆*ubp16* but not ∆*ubp8*, suggesting distinct roles for these DUBs. Nucleosome positioning analyses revealed that, following stress, nucleosomes at stress-responsive genes returned to their original positions in wild-type cells, whereas ∆*ubp8* and ∆*ubp16* mutants exhibited delayed chromatin reassembly, facilitating continued Pol II elongation. These findings suggest that Ubp8 and Ubp16 play distinct roles in transcription regulation, with Ubp8 acting at promoters and Ubp16 at coding sequences. Their absence delays nucleosome repositioning long after stress imposition, enhancing transcriptional robustness. Our study provides insights into how H2Bub deubiquitination influences transcription dynamics and chromatin architecture.

## Results

### Cells lacking the DUBs Ubp8 or Ubp16 were resistant to oxidative stress

**Fig. 1:**
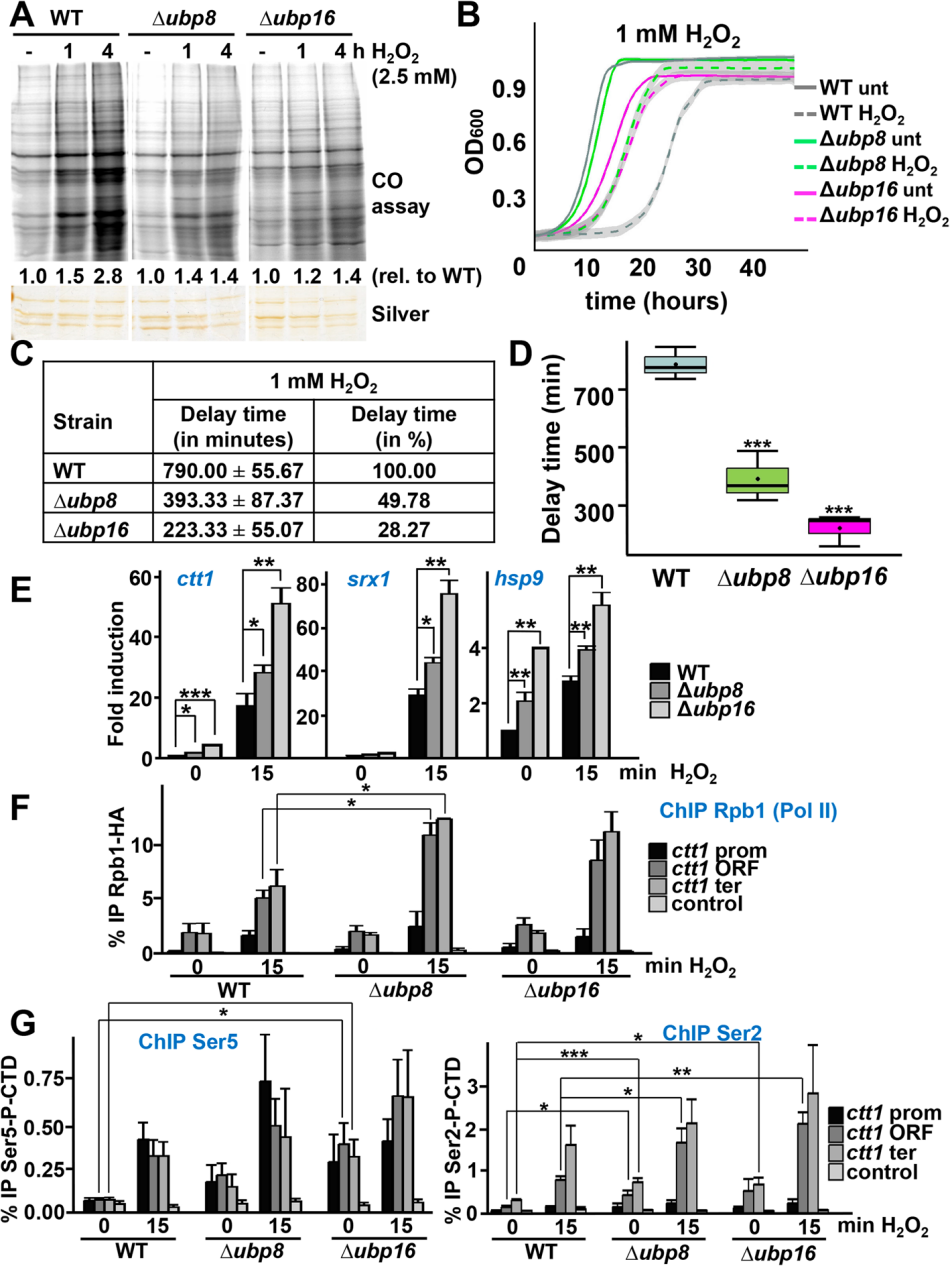
Ubp8- or Ubp16-depleted cells display resistance to oxidative stress due to enhanced stress-induced gene transcription. **A** Protein carbonyl (CO) determination of extracts from strains 972 (WT), RB64 (Δ*ubp8*) and RB65 (Δ*ubp16*) treated or not with 2.5 mM H_2_O_2_ for 1 or 4 h_._ Silver staining was used as loading control. Quantification of carbonylation levels are indicated as relativized to untreated levels. **B** Growth curves of strains as in (**A**) comparing untreated (straight lines) and treated with 1 mM H_2_O_2_ conditions (dashed lines). Each curve represents the average of three biological replicates. Shadows represent standard deviation (SD). **C** The delay in growth from (**B**) was calculated by subtracting the time required to reach an OD_600_ of 0.5 in the absence of H_2_O_2_ from the time required in its presence. Delay is represented in min, showing SD from three biological replicates, or in percentage relative to wild-type strain. **D** Boxplot representing delay times from (**C)**. ***p < 0.001. **E** Expression of the stress genes *ctt1*, *srx1* and *hsp9* under basal and treated conditions (1 mM H_2_O_2_). Total RNA from strains as in (**A**) was determined by qPCR. Amplification with *act1* primers was used as a normalization control. Fold inductions are relative to wild-type untreated, with an assigned value of 1. *p < 0.05; **p < 0.01; ***p < 0.001. **F** Pol II recruitment to stress genes. Cell cultures of strains CS61 (*rpb1-HA*), RB163 (Δ*ubp8 rpb1-HA*), and RB164 (Δ*ubp16 rpb1-HA*) were treated or not with 1 mM H_2_O_2_ for 15 min. ChIP experiments using anti-HA antibodies, coupled to qPCR, were performed using primers covering promoter, ORF, and terminator regions of *ctt1* gene. Primers of a mitochondrial DNA region were used as a negative control. *p < 0.05. **G** Cell cultures of strains as in (**A**) were treated or not with 1 mM H_2_O_2_. ChIP experiments as described in (**F**) were performed using anti-Se5-P (left panel) or anti-Ser2-P (right panel) antibodies. *p < 0.05; **p < 0.01; ***p < 0.001

Protein carbonylation is one of the hallmarks of oxidative stress toxicity. We performed a genetic screen to search for protein quality control components required for wild-type tolerance to H_2_O_2_-induced total protein carbonylation, using a collection of 74 deletion mutants lacking individual protein quality control components [32]; among them, two mutants lacking the deubiquitinases Ubp8 and Ubp16 displayed a total or partial reduction of protein carbonylation levels after peroxide stress (Fig. 1A). The absence of accumulation of oxidized proteins during H_2_O_2_ stress in these mutants could coincide with enhanced tolerance to oxidative stress. As shown in Fig. 1BCD, the time of recovery after addition of 1 mM H_2_O_2_ to liquid cultures ranged from 223 to 790 min, and was shorter in the mutants than in wild-type cultures. As often occurring in strains resistant to oxidative stress, these mutants also displayed elongated lifespan, as demonstrated by measuring viability on solid plates (Additional file 1: Fig. S1A) or by exclusion of fluorescent dyes (Additional file 1: Fig. S1B).

Ubp8 has been previously described to have H2B as a main substrate [33]. Therefore, the enhanced tolerance of these mutants to peroxides could be caused by altered transcription of the stress genes required for stress cell adaptation and survival, which are dependent on the activation by the kinase Sty1 and the transcription factor Atf1 [34]. As shown in Fig. 1E by quantitative polymerase chain reaction (qPCR), the H_2_O_2_-induced levels of the stress genes *ctt1, srx1* and *hsp9* were significantly higher in cells lacking the DUBs. In some genes such as *ctt1* and *hsp9*, basal expression was also up-regulated in these mutants. Over-activation of stress genes (Additional file 1: Fig. S1C) and decreased protein oxidation by peroxides (tested in Δubp16 in Additional file 1: Fig. S1D) in the DUB mutants was abolished in cells lacking Atf1. We conclude that transcription of the stress genes *ctt1, srx1* and *hsp9* is promoted by deletion of the DUBs.

To dismiss that our Δ*ubp8* or Δ*ubp16* strains had compensating or pleiotropic effects causing stress resistance or stress gene up-regulation, we used two strategies. Firstly, we generated degron strains by tagging the endogenous *ubp8* and *ubp16* loci with AID-coding tags [35]. While the Ubp16-AID protein levels were not altered upon auxin addition (data not shown), Ubp8-AID protein disappeared from extracts (Additional file 1: Fig. S2A), the strain became resistant to peroxides (Additional file 1: Fig. S2B) and the *ctt1* and *srx1* genes displayed stronger expression than wild-type cells upon H_2_O_2_ stress (Additional file 1: Fig. S2C). In a second approach, we generated new deletion strains by transformation (Additional file 1: Fig. S2D), and grew 5 different clones of each gene deletion to check tolerance to peroxides. As shown in Additional file 1: Fig. S2E, all the individual clones displayed shorter than wild-type time of recovery upon addition of 1 mM H_2_O_2_ to liquid cultures.

We tested whether the improved transcription of stress genes observed in cells lacking Ubp8 or Ubp16 was due to more efficient recruitment of Pol II to these genes. We chose to analyze the *ctt1* gene, which serves as a highly effective reporter of transcriptional efficiency for two main reasons. First, it exhibits one of the strongest induction responses to H_2_O_2_ (∼35-fold [34]), making it particularly sensitive to transcriptional or epigenetic disruptions [24, 36]. Second, overexpression of Ctt1 alone can compensate for defects in oxidative stress signaling pathways [37], highlighting its functional significance in H_2_O_2_ tolerance. Thus, *ctt1* not only reports transcriptional changes but also directly influences oxidative stress resistance in *S. pombe*. As shown in Fig. 1F with chromatin immuno-precipitation (ChIP), the Rpb1-HA subunit of Pol II was detected at both promoter and coding sequence of the *ctt1* gene after stress imposition, as previously reported [24], and this recruitment was more pronounced in cells lacking the DUBs. Similar results were obtained measuring phosphorylation of Pol II C-terminal domain at serine (Ser) 5 at promoters and Ser2 at ORFs and terminators of the *ctt1* stress gene (Fig. 1G and Additional file 1: Fig. S2F). These results suggest that recruitment and phosphorylation of Pol II at stress genes is facilitated by the absence of the DUBs.

### The transcriptional response to stress was robust in Δ*DUB*s strains

**Fig. 2:**
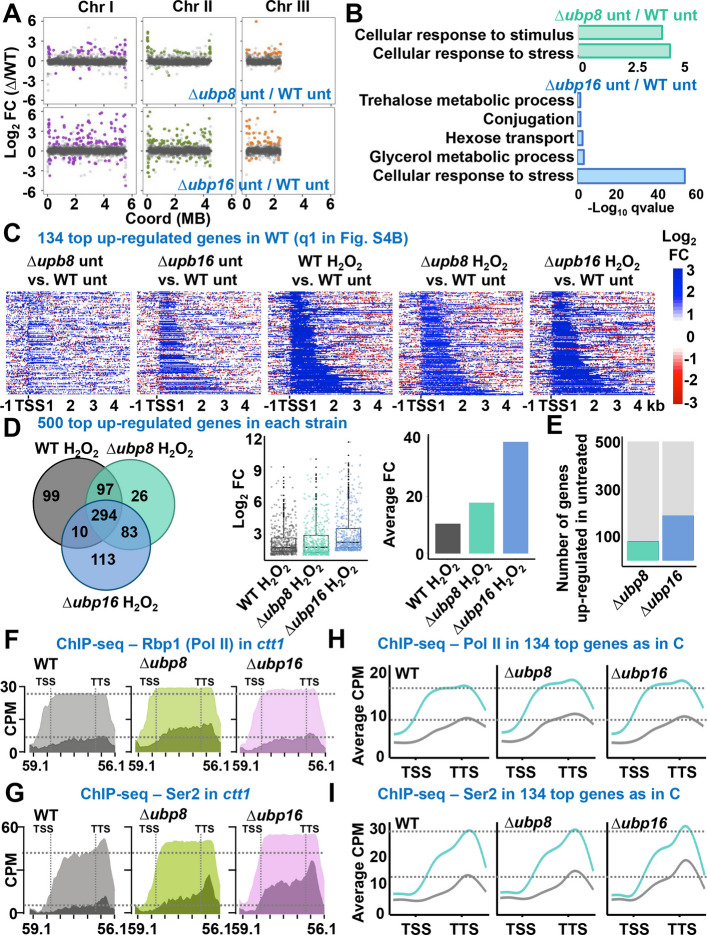
Transcriptome and Pol II landscape of DUB mutants showed up-regulated stress response. **A** Scatter plot showing in the y-axis the Log_2_ Fold Change of DESeq2 normalized reads of RB64 (Δ*ubp8*) (three upper panels) and RB65 (Δ*ubp16*) strains (three lower panels) relative to the 972 (WT) strain. X-axis represents the mid coordinate position of each *S. pombe* gene, represented with one dot, at chromosome I (left panels), chromosome II (central panels), and chromosome III (right panels). Significantly up-regulated and down-regulated genes at chromosomes I, II and III are painted in purple, green and orange, respectively. **B** GO process enrichment of significantly up-regulated genes in Δ*ubp8* or Δ*ubp16* strains under untreated conditions relative to wild-type untreated. X-axis represents the -Log_10_ of the q.value of each GO term. Only representative GO terms are shown. **C** Heatmaps showing the expression of the most expressed genes in a WT strain after 1 mM H_2_O_2_ (q1 genes from figure S4B). Log_2_ Fold Changes of normalized coverage of the indicated strains and conditions relative to WT. Genes are aligned to the TSS, and genomic regions from −1 kb to + 5 kb from the TSS are represented. Genes are arranged by length. **D** Representation of the 500 most up-regulated genes in the indicated strains after peroxide treatment. Venn diagram indicating common up-regulated genes in the three strains (left panel). Box plot represents the Log_2_ Fold Change of DESeq2 normalized reads of the most 500 expressed genes in each indicated strain relative to the WT untreated cells (central panel). Average Fold Change expression of genes in panel (**B**) (right panel). **E** Bar plots represent the number of genes among the 500 most induced genes in each strain [genes in panel (**D)**] that are already significantly up-regulated in untreated conditions in RB64 (Δ*ubp8*) and RB65 (Δ*ubp16*). **F** ChIP-seq occupancy of Pol II at *ctt1* gene. Coverage of Rpb1-HA at the *ctt1* gene from strains CS61 (*rpb1-HA*), RB163 (Δ*ubp8 rpb1-HA*) and RB164 (Δ*ubp16 rpb1-HA*)**,** before and after 15 min of 1 mM H_2_O_2_. Y-axis shows count per million (CPM) normalized coverage and x-axis genomic the *S. pombe* genomic coordinates. Dark shades: untreated, light shades: H_2_O_2_-treated. **G** ChIP-seq occupancy of Pol II phosphorylated at Ser2 at *ctt1* gene. Represented as in (**F**). **H** ChIP-seq occupancy of Pol II at the 134 most upregulated stress genes shown in (**C**). Line plots represent the average CPM coverage. Gene bodies are scaled to 1 kb and aligned to their TSS. The x-axis spans from −500 bp to + 500 bp relative to the TSS and TTS, respectively. Grey lines: untreated; blue lines: H_2_O_2_-treated. **I** ChIP-seq occupancy of Pol II phosphorylated at Ser2 of the 134 most upregulated stress genes shown in (**C**). Line plots are represented as in (**H**)

In response to environmental changes, complex gene expression programs are activated, mostly at the transcriptional level. Specifically, in response to H_2_O_2_ more than 500 genes are up-regulated > two-fold in a Sty1-dependent manner [34]. We analyzed the transcriptome of wild type and DUB mutants by RNA sequencing (RNA-seq). Briefly, we isolated RNA from cell cultures of wild-type and Δ*ubp8* and Δ*ubp16* strains grown in rich media, treated or not with H_2_O_2_ (1 mM for 30 min), and performed RNA-seq of two highly reproducible biological replicates of each biological condition (Additional file 1: Fig. S3A). Basal gene expression was affected in the mutants, with 100/212 genes up-regulated and 14/69 down-regulated more than > two-fold in Δ*ubp8* and Δ*ubp16* strains, respectively (Additional file 1: Fig. S3B). In both mutants, many more genes were up-regulated than down-regulated at the basal level, with apparent random distribution along the three *S. pombe* chromosomes (Fig. 2A). More genes were constitutively up-regulated in Δ*ubp16* than in Δ*ubp8* strains, and the functional categorization based on GO enrichment analysis highlighted the dysregulation of genes in the stress, carbon metabolism, cellular response to stimulus categories (Fig. 2B). It has been reported that cells expressing the non-ubiquitinable mutant histone H2BK119R exhibit a global de-repression of antisense transcription, with ~ 4,000 antisense transcripts significantly up-regulated [8] (Additional file 1: Fig. S3C). We tested whether the opposite effect on antisense transcription could occur in cells lacking the DUBs; as shown in Additional file 1: Fig. S3C, the levels of the antisense transcripts were barely altered in Δ*ubp8* and Δ*ubp16* strains.

To study the accumulation of stress transcripts upon H_2_O_2_ treatment in the mutants, we calculated fold-inductions of the H_2_O_2_-treated Δ*ubp8* and Δ*ubp16* samples relative to the untreated wild-type values, since many of the stress genes were already up-regulated at the basal level, as shown above. We ordered the ~ 5,000 *S. pombe* coding genes in four quartiles based on gene expression upon peroxides in the wild-type background (Q1 to Q4), and showed the heatmaps of gene activation aligned to the TSS, ordered by gene size (Additional file 1: Fig. S3D). No significant differences were noticeable among the three strains regarding induction and types of activated genes. Confirming the GO analysis of the genes constitutively up-regulated in the DUB mutants (Fig. 2B), 41% (Δ*ubp8*) and 62% (Δ*ubp16*) of these genes were up-regulated upon H_2_O_2_ stress in a wild-type background (Additional file 1: Fig. S3E). Regarding transcriptional repression, Q4 genes in wild-type cells (in red in Additional file 1: Fig. S3D) were not so strongly repressed in cells lacking Ubp8 or Ubp16, as indicated by the lighter red color in the heatmaps. In fact, the fold average repression of the 538 genes repressed more than two-fold in a wild-type strain upon H_2_O_2_ treatment was 0.11, while repression went down only to 0.22 and 0.78 in Δ*ubp8* and Δ*ubp16*, respectively, for these same genes (Additional file 1: Fig. S3F). This suggests that cells with elevated levels of ubiquitinated H2B may be more resilient to transcriptional repression by H_2_O_2_.

We represented the induction of the 535 top genes of Q1, which are the genes activated > two-fold in wild-type cells, relative to the expression under unstressed conditions of the wild-type strain; the levels of basal and stressed conditions for the DUB mutants are shown (Additional file 1: Fig. S4A). The basal expression levels were slightly higher, especially in cells lacking Ubp16. This basal de-repression of stress genes in Δ*ubp16* cells could be readily observed by representing the top 134 stress genes (first quartile of genes activated > two-fold in wild-type cells; Fig. 2C). Antisense transcription of the stress genes was not enhanced in a wild-type background, and the same was observed for cells lacking the DUBs (Additional file 1: Fig. S4B).

We then focused on the genes up-regulated in cells lacking DUBs by H_2_O_2_, regardless of whether they had been expressed or not in the wild-type background. Many more genes were activated upon stress more than two-fold in Δ*ubp16* strain that in the other backgrounds (788 genes in cells lacking Ubp16 *vs.* 535 and 526 genes in wild-type and Δ*ubp8* strains, respectively). We then compared the top 500 (Fig. 2D) and top 100 (Additional file 1: Fig. S4C) genes up-regulated in each background. As shown with Venn diagrams in the left panels of Fig. 2D and Additional file 1: Fig. S4C, these sets of genes were highly overlapping among strains. Importantly, the average fold induction of the top 500 genes (Fig. 2D, center and right panels) or 100 genes (Additional file 1: Fig. S4C, center and right panels) was significantly higher in Δ*ubp8* and Δ*ubp16* cells than in a wild-type background; the effect was significantly more pronounced for the 100 most highly expressed genes (see Additional file 1: Fig. S4C vs. 2D). In conclusion, cells lacking Ubp8 or Ubp16 and probably displaying higher levels of ubiquitinated H2B triggered a stronger antioxidant gene expression program than wild-type cells.

We also observed that a high percentage of the genes up-regulated by stress in cells lacking the DUBs were already up-regulated in these strain backgrounds under untreated conditions (Fig. 2E and Additional file 1: Fig. S4D). For instance, 44 and 85 of the top 100 genes expressed by H_2_O_2_ in the Δ*ubp8* and Δ*ubp16* mutants, respectively, were already up-regulated under basal conditions in these backgrounds (Additional file 1: Fig. S4D), suggesting that the enhanced basal expression was priming these stress genes for further signal-induced activation.

We performed ChIP sequencing (ChIP-seq) analysis of Pol II and elongating Pol II phosphorylated at Ser2, to confirm that the transcriptional machinery accumulated at the 535 stress genes (those induced more than two-fold by H_2_O_2_; Additional file 1: Fig. S4A) in wild-type and in cells lacking the DUBs upon stress (Figure S5AB). Pol II subunit Rbp1 and phosphorylated Ser2 were enriched at the *ctt1* (Fig. 2FG), *srx1* and *hsp9* (Additional file 1: Fig. S5CD) stress-response genes following stress imposition; as shown above with ChIP, this recruitment was slightly more pronounced in cells lacking the DUBs, both at basal and induced conditions, in the three genes. We also averaged the presence of Pol II and phosphorylated Ser2 before and after stress in the presence or absence of the DUBs in the 134-top stress genes (q1 quartile in Additional file 1: Fig. S4B). The metaplots shown in Fig. 2HI demonstrated a larger occupancy of Pol II and its elongating Ser2 mark in Δ*DUBs* than in wild-type genes upon stress. From these experiments, we conclude that Δ*ubp8* and Δ*ubp16* cells triggered a stronger Pol II- and H_2_O_2_-dependent gene expression program than wild-type cells.

### The stress resistant phenotype of cells lacking the DUBs was dependent on H2B ubiquitination

**Fig. 3:**
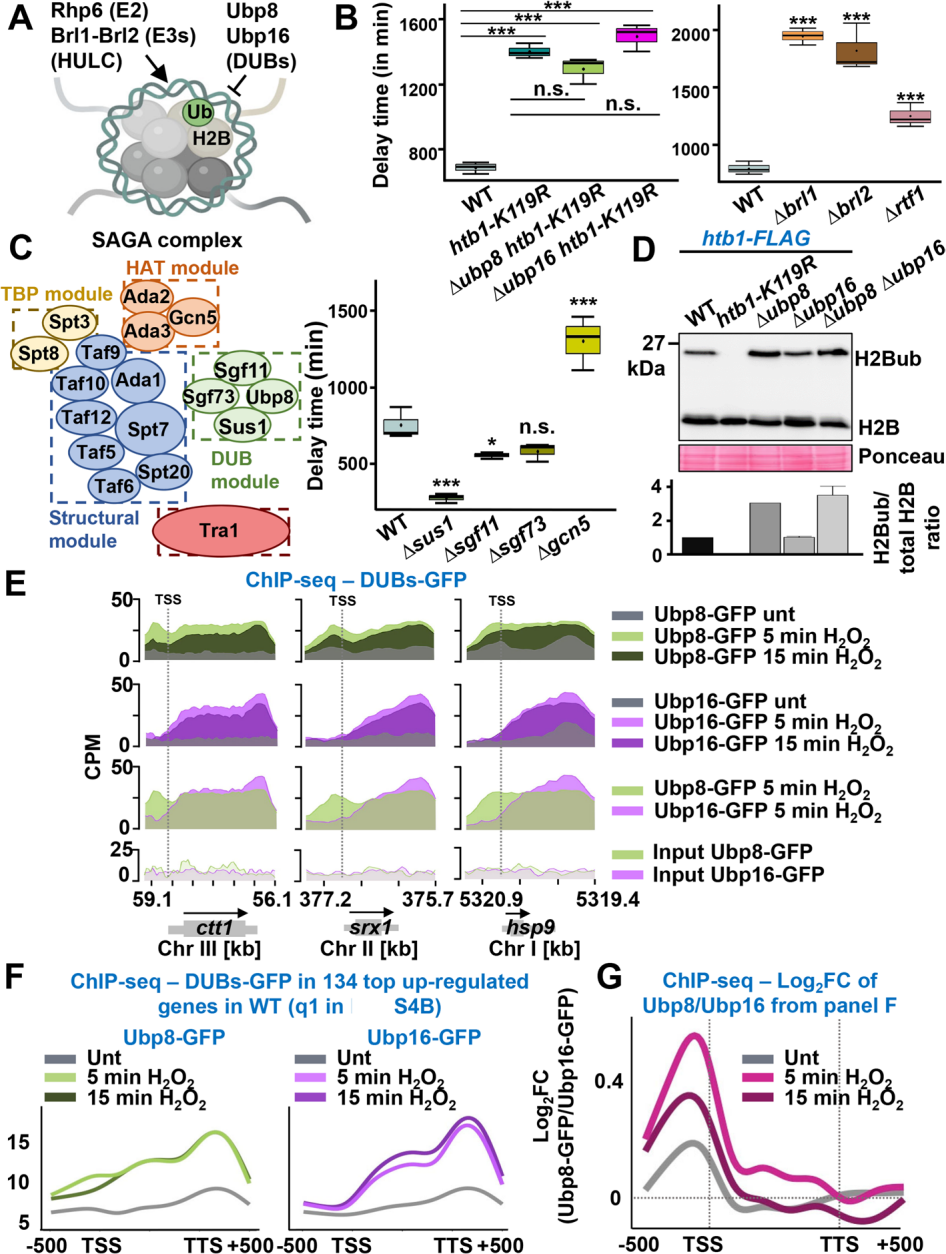
Ubp8 and Ubp16 recruited to stress genes regulate their transcription through histone H2B deubiquitination. **A** Schematic representation of participants in histone H2B ubiquitination regulation. See text for detail. Graphical scheme was created with BioRender. **B** H2Bub mutants show sensitivity to peroxides. Boxplots indicating delay times of 972 (WT), RB201 (htb1-K119R), RB100 (Δubp8 htb1-K119R) and RB101 (Δubp16 htb1-K119R) in the left panel; and 972 (WT), RB158 (Δbrl1), RB204 (Δbrl2), and RB191 (Δrtf1) in the right panel. ***p < 0.001; n.s., non significative. **C** Schematic representation of the SAGA complex modules and its subunits in S. pombe (left panel). Boxplots of 972 (WT), RB166 (Δsus1), RB187 (Δsgf11), RB192 (Δsgf73) representing delay times as in Fig. 1D (right panel). *p < 0.05; ***p < 0.001; n.s., non-significative. **D** TCA extracts from cell cultures of strains KGY15253 (WT)and KGY15388 (htb1-K119R), RB88 (Δubp8), RB89 (Δubp16), RB117 (Δubp8 Δubp16) carrying an endogenous htb1-FLAG locus. Western blot using polyclonal antibody anti-FLAG differentiates unmodified H2B and H2Bub, which migrates to a lesser extent in an electrophoresis gel. Ponceau was used as loading control. Bar plot below show quantification of the western: for each strain, H2Bub levels relative to total H2B (the sum of both non-ubiquitinatedH2B and H2Bub) were compared to H2Bub/H2B in wild-type cells (with an assigned value of 1). Each bar represents the mean value and SD from two biological replicates. **E** ChIP-seq occupancy of DUBs-GFP. Coverage of Ubp8-GFPand Ubp16-GFP at the ctt1 (left panels), srx1 (middle panels) and hsp9 (right panels) before and after 5 or 15 min of1 mM H2O2 treatment. Y-axis shows CPM normalized coverage and x-axis genomic S. pombe coordinates. **F** ChIP-seq occupancy of Pol II at the 134 most upregulated stress genes shown in Fig. 2C. Line plots represent the average CPM coverage of Ubp8-GFP (left panel) and Ubp16-GFP (right panel) under untreated, 5 or 15 min after 1 mMH2O2. Line plots are represented as in Fig. 2H. **G** Line plot representing Log2 Fold Change of Ubp8-GFP coverage relative to Ubp16-GFP (same subset of genes displayed in **F**)

To confirm that the role of Ubp8 and Ubp16 at stress genes was dependent on H2B ubiquitination, we tested the tolerance to peroxides of mutants of HULC (containing the E3 ligase and E2 conjugating subunits required for ubiquitination of the histone) and of cells expressing only H2BK119R (Fig. 3A). As shown in Fig. 3B and Additional file 1: Fig. S6A, cells lacking the E3 ligase subunits Brl1 or Brl2 or expressing H2BK119R were severely sensitive to the addition of H_2_O_2_. In fact, the resistance phenotype of cells lacking the DUBs was suppressed by the allele *htb1-K119R* (Fig. 3B and Additional file 1: Fig. S6A). Depletion of the transcription factor Rtf1, required to recruit HULC to chromatin, also rendered cells sensitive to peroxide stress (Fig. 3B).

The SAGA complex contains at least two histone modifiers: Gcn5 and Ubp8. As in budding yeast, the DUB module of SAGA includes the proteins Ubp8, Sgf11, Sus1 and Sgf73 (Fig. 3C, left panel). The group of Gould demonstrated that not only Upb8 but also the other three subunits of the DUB module can suppress some cells cycle defects through H2B ubiquitination [33], and we showed here that cells lacking Ubp8 (Fig. 3D) or Sus1 (Additional file 1: Fig. S6B) displayed higher levels of H2Bub and enhanced expression of signal-induced stress genes (Fig. 1E and Additional file 1: Fig. S6C). In a similar manner, and in contrast to the sensitivity observed in Δ*gcn5* cells, mutants deficient in each of these three subunits of the DUB module exhibited greater tolerance to H₂O₂ stress compared to wild-type cells (Fig. 3C, right panel and Additional file 1: Fig. S6D).

### During stress, Ubp8 was recruited to promoters of stress genes, while Ubp16 was localized at coding sequences

Ubp16 has not been described before to have H2B as a substrate in fission yeast. Unlike its *S. cerevisiae* ortholog Ubp10, depletion of Ubp16 did not seem to affect the total levels of H2Bub, as determined by Western blot, even when combined with the absence of Ubp8 (Fig. 3D). However, we confirmed that this was due to some buffering regulation in the constitutively deleted strain: the H2Bub levels of newly isolated clones of Δ*ubp16* (Additional file 1: Fig. S2E) were clearly higher than in a wild-type background (Additional file 1: Fig. S6E).

To test whether the DUBs were or not recruited to stress genes, we tagged the endogenous Ubp8 and Ubp16 with GFP or V5 at their C-terminal domain, and determined that cells expressing these tagged versions had wild-type tolerance to H_2_O_2_ in liquid cultures (Additional file 1: Fig. S6F). We used the GFP chimeras to confirm the nuclear localization of both DUBs (Additional file 1: Fig. S6G). Then, we tested by chromatin ChIP the recruitment upon stress of the V5-tagged DUBs to the promoter, ORF and termination sequences of the *ctt1* (Additional file 1: Fig. S6H) and *gpd1* genes (Additional file 1: Fig. S6I). Ubp8 was quickly enriched at the promoters of stress genes after stress, similar to what was described before for Gcn5 [24] (Additional file 1: Fig. S6H and Additional file 1: Fig. S6I, left panels). On the contrary, Ubp16 was mainly enriched at ORFs and terminators of the *ctt1* and *gpd1* genes (Additional file 1: Fig. S6H and Additional file 1: Fig. S6I, right panels).

We performed ChIP-seq analysis of DUBs-GFP to examine differences in the genome-wide distribution of the two DUBs (Additional file 1: Fig. S7A). The results confirmed that Ubp8-GFP -but not Ubp16-GFP- was enriched at the promoters of the *ctt1*, *srx1*, and *hsp9* stress-response genes following stress imposition (Fig. 3E). Ubp8 was also detected across ORFs and termination sites (as shown before for the Ubp8-containing SAGA complex [24, 25]), while Ubp16 showed specific enrichment at ORFs and termination sites (Fig. 3E). To further support this differential promoter association, we analyzed average DUB occupancy across the 535 stress genes induced more than two-fold by H_2_O_2_ (divided in four quartiles, q1 to q4, in Additional file 1: Fig. S4B). This analysis revealed strong recruitment of Ubp8 to promoters, while Ubp16 was largely absent from these regions (Fig. 3FG for the top q1 quartile; Additional file 1: Fig. S7B for q2-q4 quartiles).

Concomitant to the positioning of the DUBs at stress genes upon H_2_O_2_ addition, we detected a transient enrichment of H2Bub relative to total H2B upon stress imposition along the *ctt1* gene in wild-type cells, which was enhanced and more sustained at promoters (Δ*ubp8*) and coding sequences (Δ*ubp16*) in the DUB mutants (Additional file 1: Fig. S7C). These results suggest that the DUBs may be recruited by the H2Bub mark to eliminate it, and that chromatin at stress genes exhibits higher levels of H2Bub at promoters and coding regions in the absence of Ubp8 and Ubp16, respectively, which may facilitate Pol II progression during stress.

### Levels of H3K4me were elevated in the DUB mutants, whereas H3 acetylation remained unchanged

**Fig. 4:**
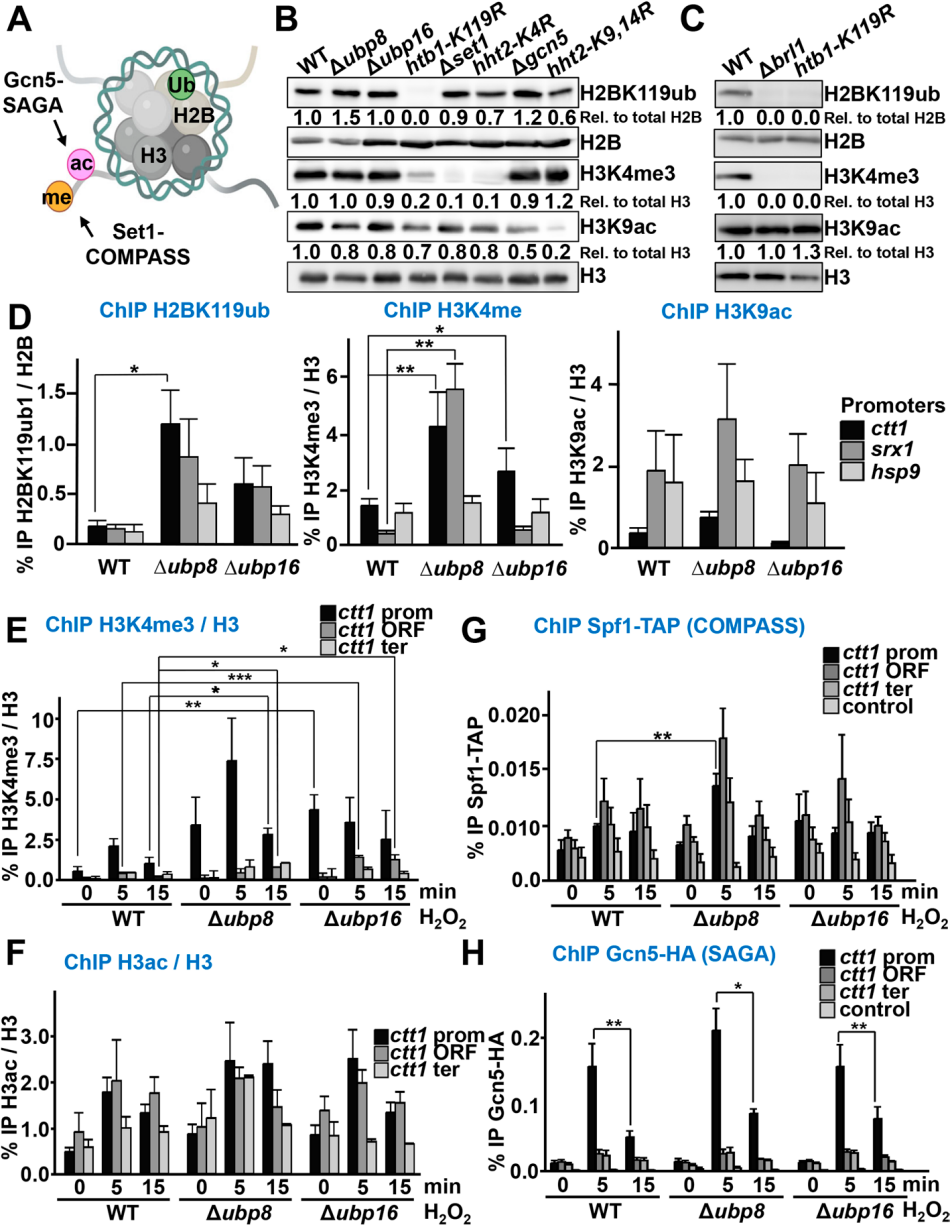
H2BK119 ubiquitination settles H3K4 methylation, but not H3 acetylation. **A** Schematic visualization of theH3K4me and H3K9ac writers, COMPASS and SAGA complex, respectively, in the histone tail of histone H3. Graphical scheme was created with BioRender. **B** TCA extracts from cell cultures of strains 972 (WT), RB64 (Δubp8), RB65(Δubp16), RB203 (htb1-K119R), JE28 (Δset1), JA1340 (hht2-K4R), MS112 (Δgcn5), and JT561 (hht2-K9RK14R). Indicated antibodies against specific histone motifs were used. Quantification of each histone mark was performed as described in Fig. 3D. **C** TCA extracts from cell cultures of strains 972 (WT), RB158 (Δbrl1) and RB203 (htb1-K119R) strains. Same antibodies as in (**B**) were used. Quantification of each histone mark was performed as described in Fig. 3D. **D** Lacking Ubp8 or Ubp16 affect histone modification at promoters of stress-genes. Cell cultures of strains 972 (WT),RB64 (Δubp8) and RB65 (Δubp16) were collected in untreated conditions. ChIP experiments were performed as Fig. 1F, coupled to qPCR, using primers covering promoter regions of ctt1, srx1, and hsp9 genes. Immunoprecipitation percentage of modified histones versus total histones is indicated. *p < 0.05; **p < 0.01; no asterisk: non-significative. **E** Methylation of H3K4 is increased at stress promoters in the DUBs mutants. Cell cultures of strains as in (**D**) were treated or not with 1 mM H2O2 at indicated times. ChIP experiments were performed, coupled to qPCR, usingsame primers as in Fig. 1F. The percentage of immunoprecipitated H3K4me3 versus total H3 is indicated. *p < 0.05;**p < 0.01; *** *p* < 0.001. **F** Cell cultures of strains as in (**D**) were treated or not with 1 mM H2O2 at indicated times. ChIP experiments were performed, coupled to qPCR, using same primers as in Fig. 1F. Percentage of immunoprecipitated H3ac versus total H3 is indicated. No asterisk: non-significative. **G** COMPASS complex recruitment in DUB mutants after stress. Cell cultures of strains RB211 (spf1-TAP), RB214 (Δubp8 spf1-TAP) and RB215 (Δubp16 spf1-TAP) were treated or not with 1 mM H2O2 at indicated times. ChIP experiments using anti-IgG, coupled to qPCR, using same primers as Fig. 1F. Four biological replicates are represented. **p < 0.01. **H** SAGA complex recruitment after stress. Cell cultures of strains MP8 (gcn5-HA), RB216 (Δubp8 gcn5-HA), and RB217 (Δubp16 gcn5-HA) were treated or not with 1 mM H2O2 at indicated times. ChIP experiments using anti-HA, coupled to qPCR, using same primers as Fig. 1F. *p < 0.05; **p < 0.01

Histone crosstalk refers to the interactions between different histone PTMs and the complexes responsible for introducing additional marks or other chromatin regulators. We and others have demonstrated that transcription regulation of stress genes by activated Atf1 requires the acetylation of histones (H3K9ac, among other residues [26]) by the Gcn5 acetyl transferase of the SAGA complex [24, 38, 39], as well as the methylation of H3 at K4 (depletion of components of Set1-COMPASS, which introduces the H3K4me mark, renders cells sensitive to peroxides [40]). We investigated the interdependence between H2Bub and the two activating marks H3K9ac and H3K4me (Fig. 4A). We first assessed the global levels of these three histone marks (H2Bub, H3K4me, and H3K9ac) by Western blot analysis in mutants lacking their respective writers (Brl1, Set1, or Gcn5) or expressing histone variants devoid of the specific lysine residue targeted by the PTM (*htb1-K119R, hht2-K4R* or *hht2-K9,14R,* respectively). As shown in Fig. 4BC, in the absence of Brl1 or in cells expressing H2BK119R, the total levels of H2Bub dropped to undetectable levels. Similarly, the absence of Set1 or expression of H3K4R resulted in undetectable levels of H3K4me in total extracts. Gcn5 is not the only histone acetyl transferase of H3K9, and therefore the levels of the histone mark H3K9ac were not fully depleted in strain Δ*gcn5*. Importantly, a unidirectional interdependence between two histone marks was observed: in the absence of Brl1 or H2BK119, the global levels of H3K4me were nearly depleted (Fig. 4BC). In conclusion, each of the marks was completely eliminated from extracts when its respective writer or the specific histone residue was absent, and total levels of histone H3K4 methylation were also dependent on histone H2B ubiquitination.

We then tested whether this histone crosstalk between H2Bub and H3K4me could be observed at stress genes. The lack of the DUBs moderately (Δ*ubp16*) of severely (Δ*ubp8*) enhanced basal levels of H2Bub at stress promoters (Fig. 4D, left panel). DUB depletion also constitutively triggered H3K4 methylation (Fig. 4D, central panel), but did not significantly affect H3K9 acetylation (Fig. 4D, right panel). Regarding stress imposition, in wild-type cells both H3K4me and H3ac were transiently enhanced at promoters of the *ctt1* gene, and also at coding sequences in the case of histone acetylation (Fig. 4EF) [24]. While the pattern of stress-dependent H3ac at the *ctt1* gene was indistinguishable between wild-type and Δ*ubp8* or Δ*ubp16* backgrounds (Fig. 4F), the levels of H3K4me were significantly elevated and sustained in cells lacking the DUBs (Fig. 4E). Concomitantly, the recruitment of COMPASS, as shown by ChIP of its Spf1(-TAP) subunit, was more pronounced in the absence of DUBs (Fig. 4G), while no significant differences were observed in the recruitment dynamics of the SAGA subunit Gcn5-HA to the *ctt1* gene (Fig. 4H). We conclude that H2Bub levels directly impact H3K4me but do not significantly affect histone H3 acetylation.

### The chromatin structure at the coding region of the stress gene *ctt1* remained open for an extended period after stress imposition in DUB mutants

**Fig. 5:**
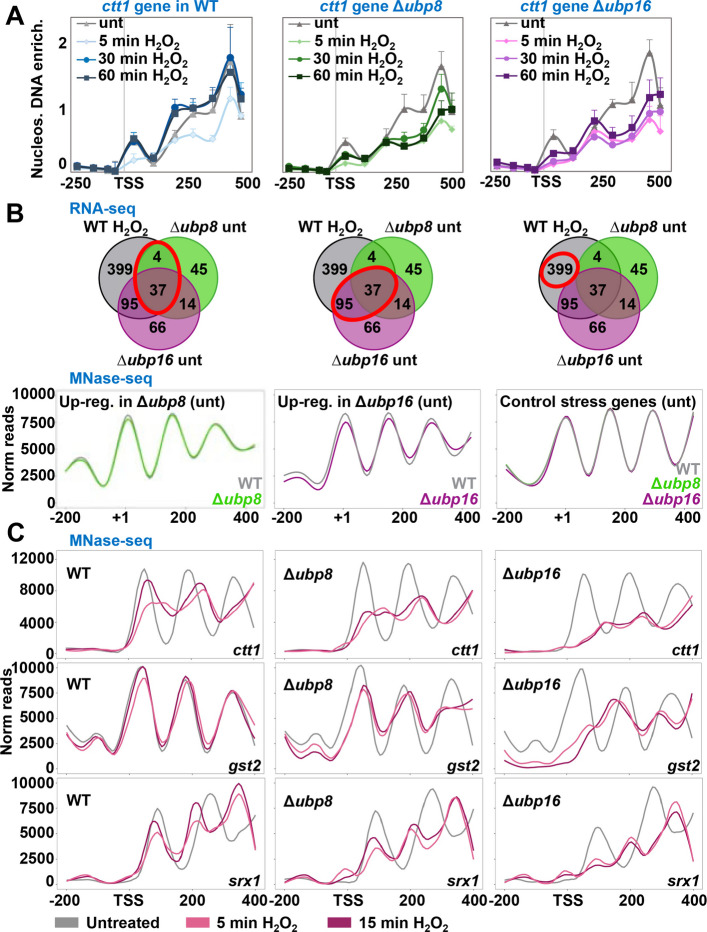
Elevated H2Bub levels alter chromatin structure. **A** Nucleosome scanning of the *ctt1* gene before (triangles) and after (5 min, diamonds; 30 min, circles; 60 min, squares) 1 mM H_2_O_2_ stress imposition was performed using strains 972 (WT), RB64 (Δ*ubp8*), and RB65 (Δ*ubp16*). **B** Venn diagrams depicting common genes significantly up-regulated in RNA-seq in the indicated strains and conditions. Red circles indicate selected genes represented in the lower panels' line plots. Line plots depict MNase-seq average coverage of the selected genes. Left panel: average reads of the stress genes induced in ∆*ubp8* untreated (41 genes); central panel: average reads of stress genes induced in ∆*ubp16* untreated (132 genes); right panel: 132 control genes induced in stress but not altered in any of the mutant strains in basal conditions. Each line represents one strain: WT in gray, ∆*ubp8* in green and ∆*ubp16* in magenta. Genes were aligned to the + 1 nucleosome (first after the TSS). **C** Nucleosome occupancy of the genes *ctt1*, *gst2* and *srx1* in the indicated strains and treatments. X-axis are the coordinates from the −200 to + 500 bp from the TSS, and y-axis normalized reads

The observed enhanced levels of H2Bub and H3K4me at stress genes in cells lacking Ubp8 or Ubp16 may facilitate Pol II progression by regulating the chromatin configuration at stress genes. As reviewed in [41], the impact of H2Bub on nucleosome architecture and recruitment of chromatin remodelers is unclear. To determine whether nucleosome positions at stress genes were affected by the absence of the DUBs, we isolated mononucleosomes from wild-type, Δ*ubp8* and Δ*ubp16* cultures before and after H_2_O_2_ stress, and then PCR-amplified the samples with overlapping pairs of primers covering ~ 0.75 kb of the *ctt1* gene, as described before [24]. Again, the *ctt1* gene was strongly up-regulated upon stress, and it fell among the 134 genes more expressed upon H_2_O_2_ (q1 in Additional file 1: Fig. S4B). As previously reported, the nucleosome landscape of the *ctt1* gene was altered upon stress imposition, probably caused by the elongating transcriptional machinery (Fig. 5A, left panel: 5 min); nucleosomes + 1 to + 3 fully returned to the starting position 30 min after H_2_O_2_ stress. The + 1 to + 3 *ctt1* nucleosomes of cells lacking Ubp8 or Ubp16 also displayed a very stable position prior to stress, which was largely lost 5 min after H_2_O_2_ stress as in wild-type cells; importantly, the + 1 to + 3 nucleosomes were not back to their original positions 30 or 60 min after stress (Fig. 5A, center and right panels; quantification for nucleosome + 1 in Additional file 1: Fig. S8A). Very similar results were observed with ChIP of H3: histone levels were reduced by half upon stress, and they were fully restored at 30 min in wild-type cells, while remained with lower occupancy in the DUB mutants (Additional file 1: Fig. S8B). These experiments suggest that enhanced levels of H2Bub impair the return to a closed configuration during stress recovery, and this would explain the more sustained presence of active Pol II-Ser2 in the Δ*DUB* mutants (30 min in Additional file 1: Fig. S2F).

To confirm the effect of H2Bub in nucleosome architecture at stress genes at a whole genome level, we purified MNase-derived mononucleosomes of wild-type and DUB mutants under untreated or H_2_O_2_-treated conditions, and sequenced them. Based on the RNA-seq experiments (Fig. 2), we selected the 41 and 132 stress genes which were up-regulated at basal conditions in a Δ*ubp8* or a Δ*ubp16* backgrounds, respectively (Fig, 5B, upper panels; circled in red), and averaged their nucleosome occupancy aligned at the center of the + 1 nucleosome (+ 1 in x-axis of Fig. 5B, lower panels). Nucleosomes + 1 to + 3 were clearly positioned in both wild-type (gray) and Δ*ubp8* strains (green), but their relative occupancy was slightly lower for cells lacking Ubp8. The difference was more pronounced for the genes up-regulated in a Δ*ubp16* background (Fig. 5B, gray for wild-type and magenta for Δ*ubp16*), with a smaller number of reads in all the nucleosomes in cells lacking the DUB. The average nucleosome profile of 132 control stress genes (whose basal expression was not enhanced in the DUB mutants) was identical across the three strains (Fig. 5B, right panel).

Regarding the nucleosome profiles of specific stress genes in response to H_2_O_2_, we confirmed with MNase-seq the results obtained for the *ctt1* gene with nucleosome scanning, and demonstrated that in this and other stress genes, such as *gst2* and *srx1,* the nucleosome architecture changed more dramatically and was more sustained in the DUB mutants than in wild-type cells, with nucleosomes not returning to the starting position at 15 min (Fig. 5C). We conclude that the chromatin remained open at longer times in the DUB mutants after stress-induced transcription activation.

We also performed an unbiased identification of genes which altered nucleosome occupancy pattern upon H_2_O_2_ stress among strains, using the DANPOS2 program [42]. Briefly, DANPOS2 uses the number of reads of nucleosome sequencing to provide a quantitative analysis of nucleosome position and occupancy. 34 and 107 genes displayed a lower number of reads in the −200 to + 400 region around the TSS under treated and untreated conditions in mutants lacking Ubp8 (Additional file 1: Fig. S8C) or Ubp16 (Additional file 1: Fig. S8D), respectively. Half of these genes were induced by H_2_O_2_ in both wild-type and the respective DUB mutant (Additional file 1: Fig. S8CD, right panels), indicating that the unbiased identification of genes with poorly positioned nucleosomes using DANPOS2 overlaps with stress response genes.

**Fig. 6:**
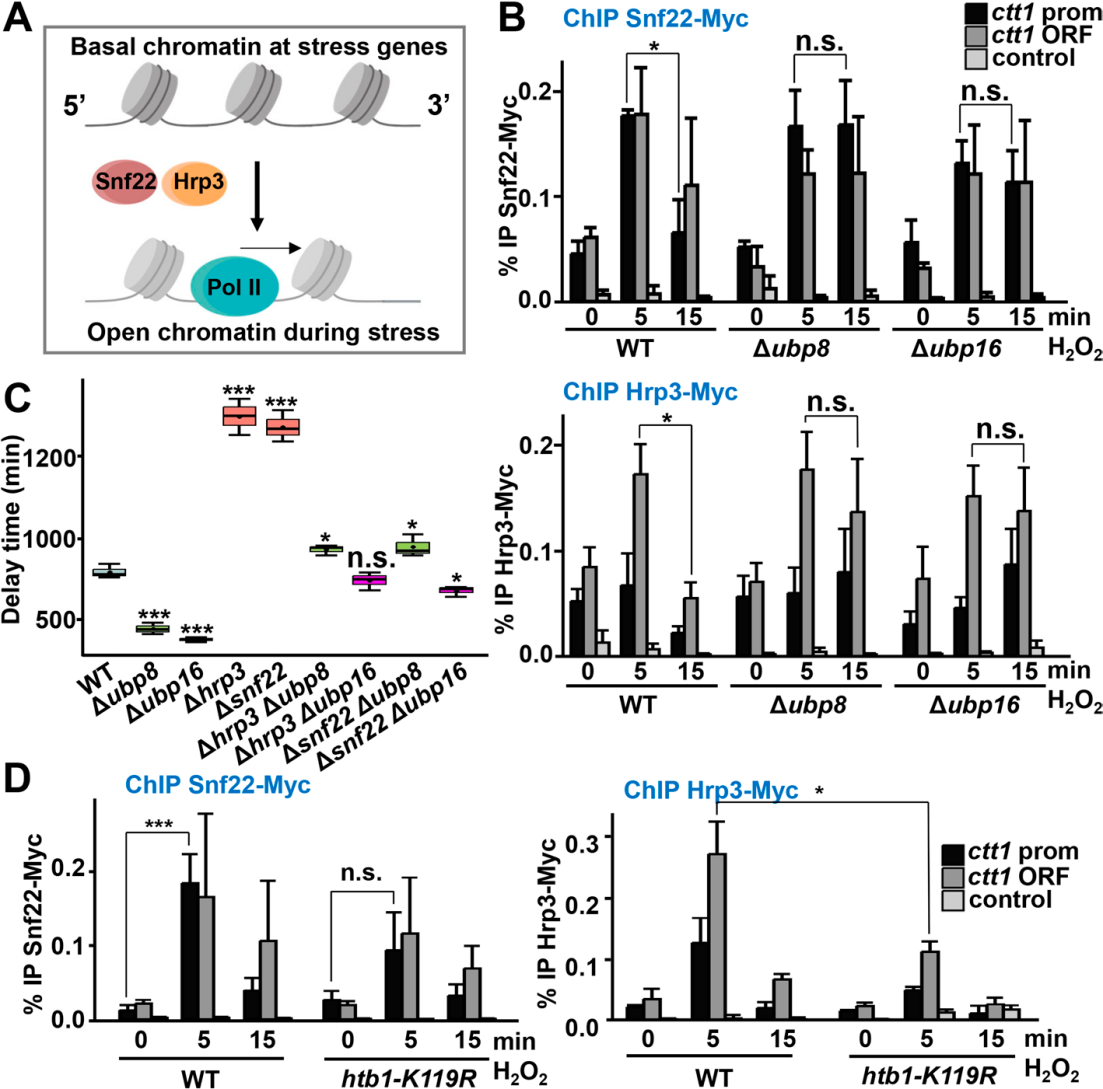
H2Bub facilitates the recruitment of chromatin remodelers at stress genes upon stress imposition. **A** Scheme depicting the proposed role of Snf22 and Hrp3 in nucleosome architecture at stress genes during early steps of transcription. Graphical scheme was created with BioRender. **B** Recruitment of chromatin remodeling proteins is prolonged in *Δubp8* and *Δubp16* cells after induction. Cell cultures of strains JA2777 (*snf22-myc*), RB195 (Δ*ubp8 snf22-myc*), and RB196 (Δ*ubp16 snf22-myc*); and JA2653 (*hrp3-myc*), RB193 (Δ*ubp8 hrp3-myc*), and RB194 (Δ*ubp16 hrp3-myc*) were treated or not with 1 mM H_2_O_2_ at indicated times. ChIP experiments using anti-myc antibodies, coupled to quantification by qPCR, were performed using primers covering promoter and ORF regions of *ctt1* gene. *p < 0.05; n.s., non-significative. **C** Genetic interactions between DUBs and chromatin remodelers. Boxplots representing delay times of strains 972 (WT), IV84 (Δ*snf22*), IV83 (Δ*hrp3*), RB199 (Δ*ubp8* Δ*snf22*), RB120 (Δ*ubp16* Δ*snf22*), RB121 (Δ*ubp8* Δ*hrp3*), RB122 (Δ*ubp16* Δhrp3) as in Fig. 1D. *p < 0.05; ***p < 0.001; n.s., non-significative. **D** Recruitment of Snf22 and Hrp3 is impaired in cells expressing H2B-K119R. ChIP analysis as in (**B**) was performed with strains JA2777 (*snf22-myc*), RB243 (*snf22-myc htb1-K119R*), JA2653 (*hrp3-myc*), and RB244 (*hrp3-myc htb1-K119R*)

We then analyzed the participation of some chromatin remodelers in the phenotypes observed in Δ*ubp8* and Δ*ubp16* strains. We recently demonstrated that the chromatin remodelers Snf22 and Hrp3 are required to open the chromatin structure of *ctt1* upon H_2_O_2_ addition, and cells lacking them are severely sensitive to oxidative stress [36] (Fig. 6A). According to ChIP analysis, while Snf22 and Hrp3 were only transiently recruited to the *ctt1* gene upon H_2_O_2_ stress in a wild-type background (5 min in Fig. 6B), their presence at DNA persisted for longer periods in cells lacking Ubp8 or Ubp16 (15 min in Fig. 6B), suggesting that they contribute to the sustained open chromatin structure in the DUB mutants. Consistently, deletion of *snf22* or *hrp3* could totally suppress the resistance phenotypes of cells lacking Ubp8 or Ubp16 (Fig. 6C and Additional file 1: Fig. S9A). In the same line, the remodelers were inefficiently recruited to DNA in cells expressing H2B-K119R (Fig. 6D).

Regarding FACT complex, the nucleosome landscape of the *ctt1* gene remained open 15 min after H_2_O_2_ stress in cells lacking the non-essential subunit Pob3 (Additional file 1: Fig. S9B), similar to what we observed in the absence of DUBs (Fig. 5A). Nevertheless, the Δ*pob3* strain was not resistant to peroxides, and strains lacking both a DUB and Pob3 displayed H_2_O_2_ resistant phenotypes (Additional file 1: Fig. S9C) and increased levels of expression of *ctt1* (Additional file 1: Fig. S9D, right panel), suggesting that elevated H2Bub levels facilitates transcription elongation even in the absence of FACT.

### H2B ubiquitination at promoters but not at ORFs can bypass the requirements of other activation marks such as H3K9ac or H3K4me

**Fig. 7:**
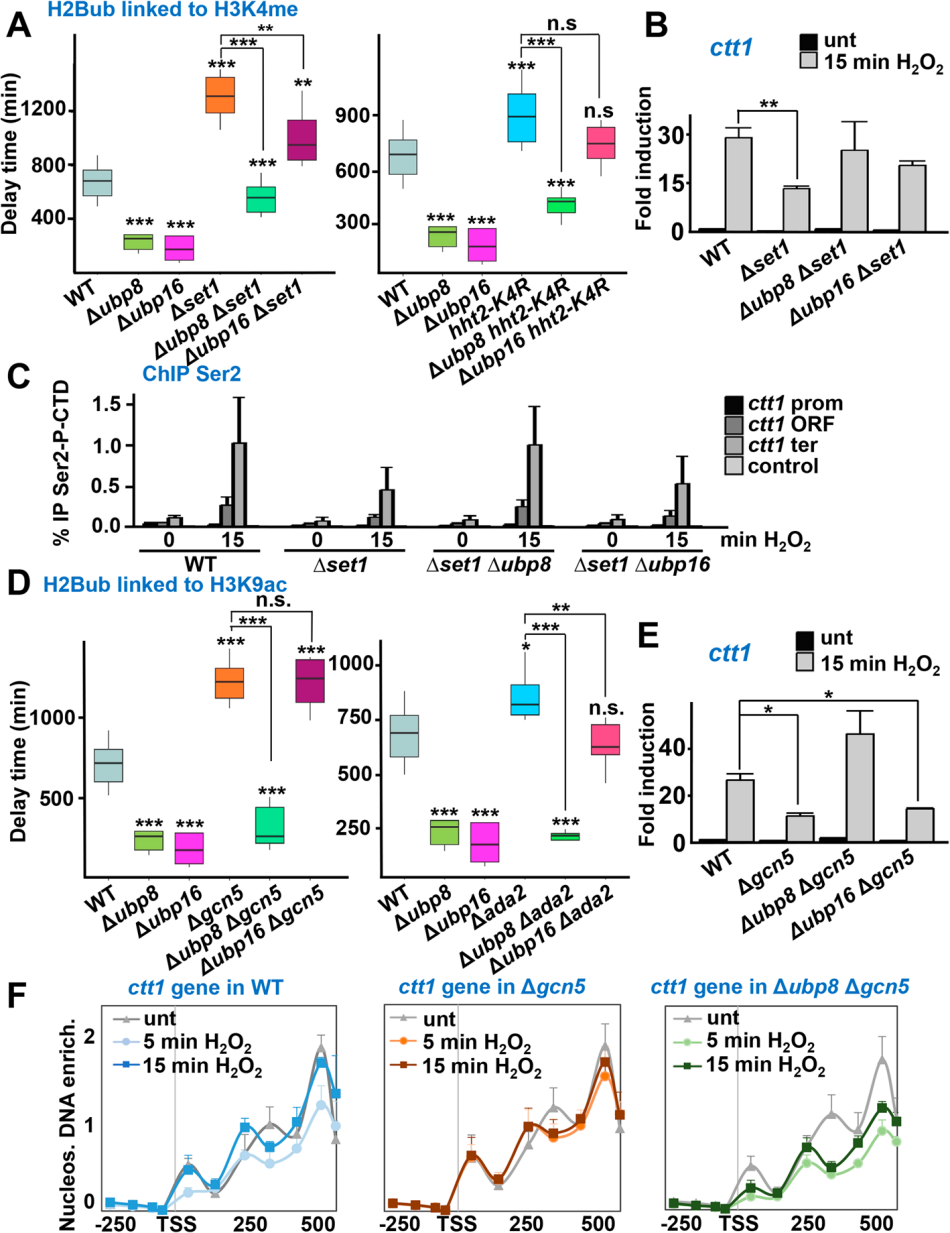
H2Bub can bypass acetylation and methylation in promoters of stress-induced genes. **A** Boxplots representing delay times, as in Fig. 1D, of strains 972 (WT), RB64 (Δ*ubp8*), RB65 (Δ*ubp16*), JE28 (Δ*set1*), RB118 (Δ*ubp8* Δ*set1*), RB119 (Δ*ubp16* Δ*set1*), JT561 (*hht2-K4R*), RB156 (Δ*ubp8 hht2-K4R*) and RB157 (Δ*ubp16 hht2-K4R*). **p < 0.01; ***p < 0.001; n.s., non-significative. **B** Relative expression of *ctt1* gene in strains as in (**A**) (left panel) was performed as in Fig. 1E. **p < 0.01. **C** Cell cultures from strains as in (**A**) were treated or not with 1 mM H_2_O_2_ for 15 min. ChIP experiments using anti-Ser2-P, coupled to qPCR, using same primers as Fig. 1F. No asterisks: non-significative. **D** Boxplots representing delay times, as in Fig. 1D, of strains 972 (WT), RB64 (Δ*ubp8*), RB65 (Δ*ubp16*), MS112 (Δ*gcn5*), RB165 (Δ*ubp8* Δ*gcn5*), RB168 (Δ*ubp16* Δ*gcn5*), IV70 (Δ*ada2*), RB177 (Δ*ubp8* Δ*ada2*) and RB178 (Δ*ubp16* Δ*ada2*). *p < 0.05; ***p < 0.001; n.s., non-significative. The control WT and single Δ*DUBs* are the same as in (**A**). **E** Relative expression of *ctt1* gene in strains as in (**D**) (left panel) was performed as in Fig. 1E. *p < 0.05. **F** Nucleosome scanning of the *ctt1* gene was performed in strains 972 (WT), MS112 (Δ*gcn5*), and RB165 (Δ*ubp8* Δ*gcn5*), represented as described in Fig. 5A

H2Bub is required for both Set1-COMPASS recruitment and activation at promoters of transcribed genes [43]. We have demonstrated that the lack of writers of three histone marks (H2Bub, H3K4me and H3K9ac) sensitizes cells to oxidative stress, and that elevated levels of H2Bub in the DUB mutants enhanced tolerance to peroxides and also caused increased levels of H3K4me. We tested whether the presence of high levels of H2Bub at promoters or at coding sequences could bypass the requirements of other histone marks. To that end, we analyzed the phenotypes of double mutants lacking the DUBs and components of the H3K4 methylation or H3K9 acetylation machineries. As shown in Fig. 7A and Additional file 1: Fig. S9E, the absence of H3K4 methylation (by expression of the H3K4R variant or in cells lacking Set1) suppressed the resistance phenotype of cells lacking Ubp16, but not of Δ*ubp8*. In the case of Δ*ubp8*, it is worth noting that the elevated levels of H2Bub triggered Set1-COMPASS recruitment and H3K4me accumulation at promoters (Fig. 4D); however, this step was not necessary for promoting stress resistance, as shown in Fig. 7A. The levels of *ctt1* gene expression (Fig. 7B) and of active transcription (as shown with ChIP of Ser2 Pol II; Fig. 7C) correlated with the observed phenotypes, with high levels in the double mutant Δ*ubp8* Δ*set1* and low levels in the Δ*ubp16* Δ*set1* strain.

In the same line, and regarding histone acetylation, Δ*ubp8* cells were still resistant to H_2_O_2_ in the absence of Gcn5 or Ada2, both essential to introduce the H3K9ac mark; on the contrary, *gcn5* or *ada2* gene deletions fully suppressed the resistant phenotype of strain Δ*ubp16* (Fig. 7D and Additional file 1: Fig. S9E). Again, the levels of *ctt1* were elevated in Δ*ubp8* Δ*gcn5* and low in the Δ*ubp16* Δ*gcn5* strain, supporting the different tolerance to peroxides (Fig. 7E).

We monitored the chromatin architecture in these double mutants. To our surprise, nucleosome occupancy in cells lacking the H3K4me writer Set1 was significantly impaired under basal conditions, and stress treatment barely induced changes in the chromatin maps (Additional file 1: Fig. S10A). To the best of our knowledge, it has not been reported before the effect of COMPASS deficiency on nucleosome positioning. Regarding the nucleosome profiles of cells lacking the H3K9ac writer Gcn5, the nucleosome positions were properly established prior to stress, and did not change upon H_2_O_2_ stress, as previously reported [24] (Fig. 7F, center panel). However, the nucleosome scanning profile of cells lacking both Gcn5 and Ubp8 were similar to those lacking only the DUB (compare Fig. 7F, right panel and Fig. 5A, center panel).

These results suggest that high levels of H2Bub at stress gene promoters, as observed in strain Δ*ubp8*, are sufficient to promote stress- and Atf1-dependent Pol II transcription even in the absence of histone acetylation or histone methylation. Conversely, the elevated levels of H2B at coding sequences in the *Δubp16* strain still rely on H3K4me and H3K9ac at promoters for transcription activation.

## Discussion

Histone PTMs profoundly affect gene expression by modulating chromatin accessibility to the transcriptional machinery. However, mutants lacking writers or erasers of these marks may exhibit long-term adaptation signatures and may display opposite effects depending on the chromosomal location being studied, complicating data interpretation. The global effect of H2B ubiquitination in Pol II-dependent transcription is controversial. Environmental stresses strongly rely on transcriptional-driven expression programs for survival, which are sharply triggered upon signal addition, and we have used this off-to-on shift of stress genes transcription as a readout of Pol II efficiency. We have investigated here how the absence of H2Bub erasers, Ubp8 or Ubp16, contributes to chromatin structure and Pol II transcription studying the H_2_O_2_ stress response. We show that the lack of DUBs partially de-represses the expression of stress genes under basal conditions, and also impairs nucleosome reassembly during stress recovery. As a consequence, cells lacking H2Bub DUBs are resistant to H_2_O_2_.

In biology, all signal-dependent processes are based on activating activities but also on inactivating ones, to return to the original state. Histone ubiquitination, likely the bulkiest histone mark, relies on two DUBs to eliminate the PTM. We show here that individual deletion of *ubp8* or *ubp16* facilitates transcription and contributes to a stress-resistant phenotype. However, the DUBs are recruited to distinct positions at transcribed genes (Ubp8 is recruited mainly at promoter regions together with SAGA, while Ubp16 is mainly localized at coding sequences), and H2Bub levels are consequently enhanced at promoters of Δ*ubp8* cells and at coding sequences of Δ*ubp16* cells even in the absence of stress. These results suggest that H2Bub has distinct functions depending on the relative gene location. At promoters in strain Δ*ubp8*, stress- and transcription factor-dependent ubiquitination of H2B at nucleosome + 1 may disrupt the stability of DNA-to-histone interactions sufficiently to promote Pol II exit from the nucleosome-depleted region at the promoter, so that acetylation and methylation are not strictly required. At coding sequences, accumulation of H2Bub in strain Δ*ubp16* may facilitate Pol II elongation, but H3K4 methylation and H3K9 acetylation of the + 1 nucleosome are still required to allow promoter escape. Regarding the role of H2Bub at promoters, this mark if indispensable for H3K4me3 at promoter-proximal positions, but it only partially reduces Set1 recruitment to these 5’ gene regions, suggesting that it may regulate both Set1-COMPASS recruitment and activity [43, 44].

We have shown before that the lack of writers of two histone marks (H3K4me and H3K9ac) sensitizes cells to oxidative stress [24, 40]. We show here that deletion of the writers of H2Bub, the HULC components Brl1 or Brl2, also renders cells with low tolerance to peroxides. Cells lacking Rtf1, which in budding yeast is required to recruit HULC [45], are also sensitive to stress, and we isolated the Paf1 complex (which couples histone modifications to efficient transcription elongation and is also required for HULC recruitment) as important to reach wild-type tolerance to H_2_O_2_ [40]. Regarding erasers, lack of the DUBs causes the opposite phenotype that the lack of writers: Δ*ubp8* and Δ*ubp16* strains are resistant to peroxides. We have not been able to report similar resistant phenotypes for cells lacking H3K4me demethylases [46] (Lsd1 or Jmj2) (Additional file 1: Fig. S10B), and depletion of only some subunits of H3K9 deacetylase complexes displayed slight enhanced tolerance (Δ*mug165,* Δ*clr3* and Δ*hos2* in Additional file 1: Fig. S10B). This highlights the very relevant role of H2B ubiquitination and deubiquitination in controlling Pol II efficiency.

Regarding histone PTMs crosstalk, we show here a clear and unidirectional interdependence between enhanced levels of H2Bub in mutants lacking the DUBs and increased H3K4me3 at promoters. The literature presents conflicting views on the global role of H3K4 methylation and transcription levels. However, our findings indicate that stress genes exhibit high levels of this mark upon stress induction, concurrent with Pol II transcription activation. This underscores the significance of using the stress response as an indicator of chromatin configuration, marked by a transition from inactive to active Pol II transcription. Consistent with our results, acute ablation of Set1-COMPASS in embryonic stem cells leads to a complete loss of H3K4 methylation, without detectable effects on transcriptional initiation but with a widespread decrease in transcriptional output and slower elongation [47].

Is the enhanced transcription efficiency observed in the DUB mutants caused by destabilization of the nucleosome architecture by elevated H2Bub? Again, previous genome-wide studies seem to contradict this theory. Using genome-wide nucleosome sequencing, the group of Pugh proposed a widespread role of H2Bub in promoting nucleosome assembly across the budding yeast genome, with negative consequences on Pol II recruitment/transcription initiation at promoters but positive consequences on Pol II elongation into the body of genes [31]. Our nucleosome scanning analysis, using the *ctt1* gene as a reporter, indicate that the chromatin remodelers Snf22 and Hrp3 likely facilitate nucleosome disassembly during Pol II elongation in the first rounds of the stress transcriptional waves; in the absence of Ubp8 or Ubp16, closing of the chromatin structure is delayed, allowing a more sustained transcriptional response. Increased H2Bub caused by lack of the DUBs seems to enhance stress gene induction and H_2_O_2_ resistance independently of either individual remodeler. Furthermore, many stress genes are up-regulated under basal conditions in the DUB mutants, and those genes display a slight decrease in nucleosome reads based on nucleosome sequencing (Fig. 5BC). Of note, the number of genes up-regulated under basal conditions and upon stress imposition are higher in cells lacking Ubp16 than Ubp8, and these features correlate with the stronger stress resistant phenotype of strain Δ*ubp16*.

Regarding the role of FACT complex in the phenotypes observed in the DUB mutants, our data does not support a straightforward correlation between H2Bub and FACT, since the improved transcription of *ctt1* and the resistance phenotype of cells lacking the DUBs do not depend on the presence of the non-essential subunit Pob3. Regarding the role of this complex on stress survival, the chromatin architecture around the *ctt1* gene in Δ*pob3* cells is reminiscent to Δ*ubp8* or Δ*ubp16* cells (nucleosomes are evicted but do not return to their original positions fast during recovery), and nevertheless strain Δ*pob3* is not resistant to stress. The role of FACT in promoting transcription is currently being unraveled. It was originally proposed that H2Bub stimulates FACT to displace H2A/H2B dimer from the transcribed nucleosomes, enhancing the frequency of Pol II passage through the nucleosomes [6]. However, at least in budding yeast, FACT and H2B may contribute both to H2A/H2B dimer displacement from the Pol II-transcribed nucleosome, and the DUB Ubp10 would then mediate dimer reposition by FACT at its hexanucleosome at the Pol II wake [30]. In *S. pombe,* it has been recently proposed that the two subunits of FACT, Pob3 and the essential Spt16, have distinct role at 5’ and 3’ end of genes, and that H2Bub enhances histone release from FACT, but it also maintains the complex within genes [8].

In summary, despite their shared substrate specificity and the common stress-resistant phenotype upon depletion, Ubp8 and Ubp16 appear to play distinct molecular roles in vivo. Ubp8 is associated with promoters of stress genes following stress imposition, while Ubp16 is predominantly localized within ORFs and terminators. Additionally, both deletion mutants exhibit increased levels of H2Bub and H3K4me, but despite these commonalities, they differ significantly in their genetic interactors. Ubp8, which is recruited to promoters along with the activating SAGA complex, may help buffer the transcriptional machinery to prevent over-activation, as the H2Bub mark seems sufficient to overcome the transcription barrier of nucleosome + 1 even in the absence of H3K9ac or H3K4me. Conversely, the elevated H2Bub at coding sequences in cells lacking Ubp16 is insufficient to enhance transcription levels in the absence of Gcn5 and Set1-COMPASS, which are required to allow Pol II promoter escape. Future studies centered on the isolation of the interactomes of these two DUBs may help us elucidate the local role of H2Bub in transcription efficiency.

## Conclusions

Our study highlights the relevance of H2Bub in transcription. Cells lacking the DUBs Ubp8 or Ubp16 display enhanced tolerance to stress thanks to the basal and induced up-regulation of stress genes. Accumulation of this histone mark at promoters in cells lacking the DUB Ubp8 is sufficient to promote Pol II transcription even in the absence of H3K9 acetylation or H3K4 methylation. In contrast, the phenotypes of strain Δ*ubp16* are suppressed by the lack of other histone writers. We believe that these findings may open the door to understanding the complex cross-talk between histone PTMs and Pol II transcription.

## Materials and methods

### Yeast strains, plasmids and growth conditions

Cells were grown in rich medium (YE5S) as described previously [48]. All yeast strains used in this study are outlined in Additional file 2: Table S1 and were constructed by standard genetic methods. Some of the strains were kindly provided or we previously generated them, as indicated [49–54]. For the application of the auxin-inducible degron (AID) system, we used the 3 × sAID degron fused to Ubp8 protein, which results in its degradation in the presence of the bulky analog of auxin, 5-adamantyl-IAA (TCI chemicals; A3390, used at 1 μM final concentration), in a OsTIR1-F74A genetic background, as described in [35].

### Protein carbonylation (CO) assay

Sample collection and preparation was performed as previously described [55]. Briefly, yeast cells were grown to an OD_600_ of 0.5, and were treated or not with 2.5 mM H_2_O_2_ for 1 or 4 h. Cells from 50 ml cultures were resuspended in carbonylation buffer and lysed. Protein extracts were incubated with streptomycin sulfate (Sigma, S6501) before incubation with fluorescein 5-thiosemicarbazide (FTC) (Sigma, 46,985). To visualize protein carbonyls, protein extracts were loaded in sodium dodecyl sulfate–polyacrylamide electrophoresis (SDS-PAGE) gels. Gels were scanned using Typhoon 8600 Variable Mode Imager scanner (Molecular Dynamics) with a 526 nm short pass filter at 800 V. Gels were then fixed and total protein was visualized by silver staining. Protein carbonyl levels were quantified using the ImageQuant 5.2 program (GE healthcare, Little Chalfont, Buckinghamshire, United Kingdom) for carbonyls and ImageJ software for total protein.

### H_2_O_2_ liquid survival assays

Assays were performed as previously described [56]. Briefly, cell cultures were grown to an OD_600_ of 0.1. Then 100 µl cultures were treated or not with 1 mM H_2_O_2_, and transferred to 96-well plates, where OD_600_ was monitored through 48 h at 30ºC using Power Wave microplate scanning spectrophotometer (Bio-Tek, Winooski, VT, USA) and Gen5 software version 1.04.5, as previously described [40].

### Solid plates assay

Assays were performed as previously described [56]. Briefly, serial dilutions of yeast cultures at the logarithmic or stationary phase (Day 2 or Day 5) were spotted on agar plates. Plates were allowed to dry and plates incubated for 2 days at 30ºC.

### Measuring chronological lifespan by flow cytometry

Cell cultures were grown in logarithmic phase and cell viability was measured every 2 days in stationary phase using propidium iodide (PI) and flow cytometry as previously described [57]. Briefly, cells were collected, resuspended and incubated with PI during 30 min at 30°C in the dark. Cells were analyzed by flow cytometry. We extracted the data of the percentage of living cells to build survival curves, considering that the logarithmic phase sample contains 100% of viable cells.

### RNA isolation and analysis by quantitative PCR (qPCR)

Cells grown to an OD_600_ of 0.5 were left either untreated or were treated for 15 or 30 min with 1 mM of H_2_O_2_. RNA isolation was performed as previously described [36]. Error bars (SD) were calculated from three biological replicates, and *act1* mRNA was used as a control for normalization. Fold induction was calculated comparing the value of each strain and condition to that of the untreated condition of the wild-type strain. Primers used for *ctt1, srx1, hsp9* and the control *act1* were described in [36].

### RNA sequencing and analysis

Total RNA from the indicated strains*,* under untreated conditions and after 30 min of 1 mM H_2_O_2_ treatment was obtained as described above. Following ENCODE guidelines, two biological replicates were analyzed (RNA-seq couples have a Spearman correlation coefficient higher than 0.98). Libraries were prepared as described in [36]. Sequencing was done using the NextSeq2000, Single Reads, and 50 nts. For the RNA-seq analysis, raw FASTQ files were first evaluated using quality control checks from FastQC 0.11.9. Alignment was done against the ‘*Schizosaccharomyces pombe* all chromosomes 2023/03/15’ reference genome using STAR 2.7.9a. Uniquely mapped reads were counted per genes in sense or antisense orientation. Quantification results were then imported to DESeq2 1.36.0 for differential expression analysis. The threshold used to identify differentially expressed genes was two-fold with a p.adjusted value lower than 0.1. Normalized coverage tracks were generated from BAM files using deepTools v3.5.1.

### Chromatin immuno-precipitation (ChIP)

Chromatin isolation and immuno-precipitation were carried out as previously described [36]. Chromatin was immuno-precipitated with specific antibody [5 μl of anti-HA antiserum (12CA5; house-made), 1 μl anti-phospho-Ser2 RNA Pol II CTD polyclonal (Abcam; ab5095), 1 μl anti-phospho-Ser5 RNA Pol II CTD polyclonal (Abcam; ab5131), 1 μl of anti-V5 monoclonal (BioRad; MCA1360), 1 μl of anti-GFP polyclonal (house-made), 1 μl of anti-H2BK119ub monoclonal (ActiveMotif; AB_2793279), 1 μl of anti-H2B polyclonal (Abcam; ab188271), 1 μl of anti-H3K4me3 polyclonal (Abcam; ab8580), 1 μl of anti-H3ac (Sigma; 06–599), 1 μl of anti-H3K9ac (Sigma; 07–352), 1 μl of anti-H3 (Millipore; ab1791), 10 μl of IgG Sepharose (Sigma; GE17-0969–01), 1 μl of anti-Myc polyclonal (SIGMA; C3956)], overnight at 4 °C rotating. Beads were washed, DNA was eluted and formaldehyde cross-linking was reversed. After protein digestion and chromatin extraction, DNA was amplified by real-time quantitative PCR using Light Cycler 480 SYBR Green I Master (Roche). Primers used for *ctt1, gpd1, srx1, hsp9* and the control *mtDNA* were described in [36]. Each column represents the mean value and SD as error bars, calculated from three biological replicates.

### ChIP sequencing (ChIP-seq)

ChIP of cultures, treated or not for 5–15 min with 1 mM H_2_O_2_, were obtained from the indicated strains as described above. Following ENCODE guidelines for ChIP-seq experiments, two biological replicates were performed and analyzed (ChIP-seq couples have a Spearman correlation coefficient higher than 0.98). DNA libraries were prepared using NEBNext® Ultra™ II DNA Library Prep Kit for Illumina, following manufacturer’s instructions, and subjected to single-end sequencing using Illumina Nextseq 2000 platform. BBDuk was used for quality and adapter trimming of reads. Reads were aligned using Bowtie2, and low-quality alignments were filtered with SAMtools. BAM file comparison, coverage normalization, and BigWig file generation were performed using deepTools.

### TCA extracts and immunoblot analysis

Sample collection was performed as previously described [36]. Extracts were separated by SDS-PAGE and detected by immunoblotting with anti-H2BK119ub monoclonal (ActiveMotif; AB_2793279), anti-H2B polyclonal (Abcam; ab188271), anti-H3K4me3 polyclonal (Abcam; ab8580), anti-H3K9ac polyclonal (Sigma; 07–352), anti-H3 polyclonal (Millipore; ab1791), anti-FLAG polyclonal (Sigma; F7425), anti-mini-AID (MBL International Corporation; M214-3).

### Mononucleosome purification and nucleosome scanning analysis

Nucleosome profiles at the *ctt1* gene were determined by nucleosome scanning as previously described [36]. Non-gel purified fractions of mononucleosomal DNA were PCR-amplified with pairs of overlapping primers covering ~ 0.8 kb of the *ctt1* gene. Pairs of primers centered at positions, relative to the TSS, of −221, −144, −77, −43, + 45, + 137, + 238, + 323, + 415, + 498 and + 548. Relative levels were addressed through amplification using *ade6* gene + 143. PCR efficiency for each primer pair was calculated with serial dilutions of template DNA (from 10^−1^ to 10^−4^). Each column represents the mean value and SD, calculated from three biological replicates.

### Mononucleosome purification and sequencing (MNase-seq)

Mononucleosome purification from the indicated strains in untreated conditions or after 5 and 15 min of H_2_O_2_ treatment was performed as above, with a final gel purification step of the mononuclesomes, as described in [36]. Following ENCODE guidelines for nuclease sequencing experiments, two biological replicates were performed and analyzed (RNA-seq couples have a Spearman correlation coefficient higher than 0.98). DNA libraries were prepared using NEBNext® Ultra™ II DNA Library Prep Kit for Illumina, following manufacturer’s instructions, and subjected to paired-end sequencing using Illumina Nextseq 2000 platform. Analysis was performed as described in [36], with around 50 million reads per sample. Briefly, reads were align using Bowtie2, SAMtools to filter low quality alignments and DANPOS2 algorithm was used remove clonal reads, adjust read length, perform quantile normalization, identify nucleosomes in the genome and calculate differences in nucleosome occupancy [42].

### Statistical analysis

Unless otherwise stated, all experiments were performed at least three times and representative ones are shown. For statistical analysis two-sided t-test was performed.

## Supplementary Information

Additional file 1: Fig. S1. Ubp8 or Ubp16 depletion confers long-lived phenotypes depending on Atf1 activity. Fig. S2. Alternative strategies to generate Ubp8 or Ubp16 depleted cells. Fig. S3-S4. Transcriptome landscape of DUB mutants showed up-regulated stress response. Fig. S5. ChIP-seq analysis of Rbp1-HA (Pol II) and phosphorylated Pol II at Ser2. Fig. S6. Mutations of H2B ubiquitination regulators affect tolerance to oxidative stress. Fig. S7. ChIP-seq analysis of Ubp8 and Ubp16. Fig. S8. Nucleosome eviction upon H_2_O_2_ treatment is more sustained in cells lacking DUBs. Fig. S9. Genetic interactions of H2Bub with chromatin remodelers, FACT, SAGA or COMPASS. Fig. S10. Chromatin remodelers, and histone marks writers genetically interact with DUBs function.

Additional file 2: Table S1. Strains used in this study.

## Data Availability

Original data such as images and blots have been deposited in Figshare (https://figshare.com/s/b9e126e2b66a4e9519d1) [58]. Regarding RNA-seq, ChIP-seq and MNase-seq, the data underlying this article are available in GEO (RNA-seq: GSE279908 [59]; ChIP-seq: GSE303160 [60]; MNase-seq: GSE279944 [61]). Published RNA-seq data from the FACT complex was also used [62]. To visualize RNA-seq, ChIP-seq and MNase-seq data in a genome browser, the bw. files deposited at GEO can be directly loaded at the JBrowse of Pombase (https://www.pombase.org/jbrowse/).
