# Supplementary material for: Distinct roles of histone H2B ubiquitination at promoters and coding regions of Pol II-transcribed stress genes
Source: Genome Biol. 2025 Dec 9;26:419. doi: 10.1186/s13059-025-03891-1 (PMC12687531; doi:10.1186/s13059-025-03891-1)
Supplement: Supplementary file 1 — Additional file 1: Fig. S1. Ubp8 or Ubp16 depletion confers long-lived phenotypes depending on Atf1 activity. Fig. S2. Alternative strategies to generate Ubp8 or Ubp16 depleted cells. Fig. S3-S4. Transcriptome landscape of DUB mutants showed up-regulated stress response. Fig. S5. ChIP-seq analysis of Rbp1-HA (Pol II) and phosphorylated Pol II at Ser2. Fig. S6. Mutations of H2B ubiquitination regulators affect tolerance to oxidative stress. Fig. S7. ChIP-seq analysis of Ubp8 and Ubp16. Fig. S8. Nucleosome eviction upon H2O2 treatment is more sustained in cells lacking DUBs. Fig. S9. Genetic interactions of H2Bub with chromatin remodelers, FACT, SAGA or COMPASS. Fig. S10. Chromatin remodelers, and histone marks writers genetically interact with DUBs function. [file 13059_2025_3891_MOESM1_ESM.pdf]

ADDITIONAL FILE 1

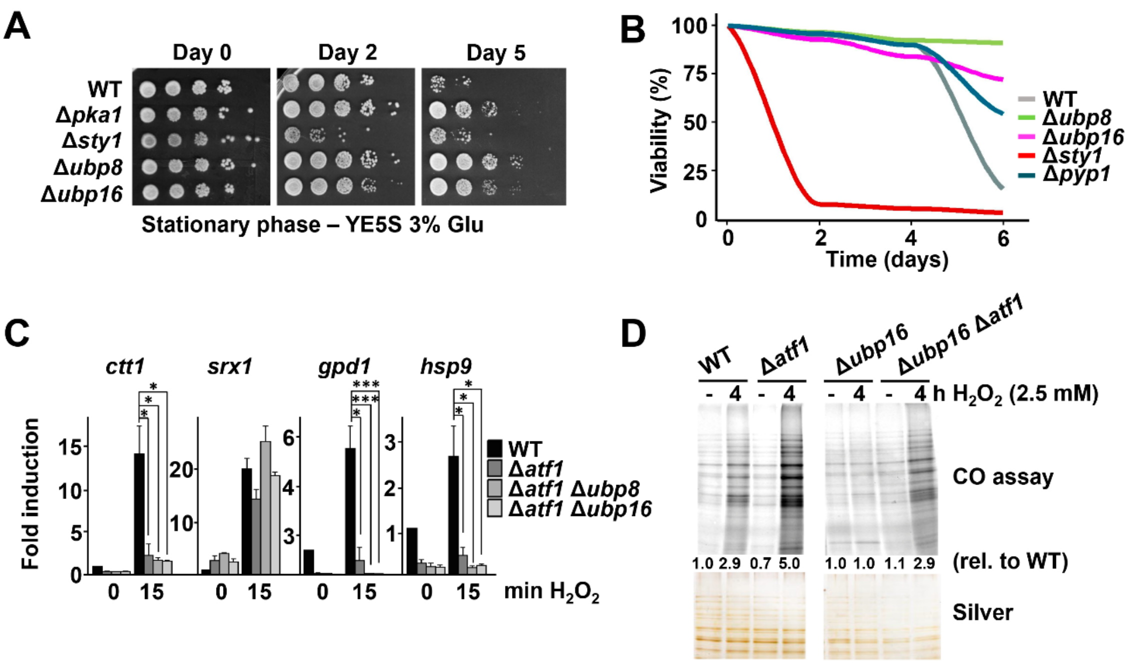

Fig. S1 Ubp8 or Ubp16 deletion confers long-lived phenotypes depending on Atf1 activity. **A** Serial dilutions of cell cultures of the strains 972 (WT), AZ74 (Δ*pka1*), AV18 (Δ*sty1*), RB64 (Δ*ubp8*), and RB65 (Δ*ubp16*) were spotted in agar plates from logarithmic (Day 0) or stationary phase (Day 2 and Day5) cultures. **B** Lifespan of 972, AZ63 (Δ*pyp1)*, AV18, RB64, and RB65 strains were measured by propidium iodide staining and FACS analysis. Line plot represents the local regression curves for the average survival of each strain at different time points. **C** Relative expression of *ctt1*, *srx1*, *gpd1* and *hsp9* were performed as in Fig. 1E for strains 972 (WT), MS98 (Δ*atf1*), SB442 (Δ*ubp8* Δ*atf1*), and SB443 (Δ*ubp16* Δ*atf1*). **p*<0.05; ****p*<0.001. **D** Protein carbonyl (CO) determination of extracts from strains 972 (WT), MS98 (Δ*atf1*)*,* RB65 (Δ*ubp16*) and SB443 (Δ*ubp16* Δ*atf1*) was performed as described in Fig 1A, after 4 hours of 2.5 mM H_2O_2 treatment.

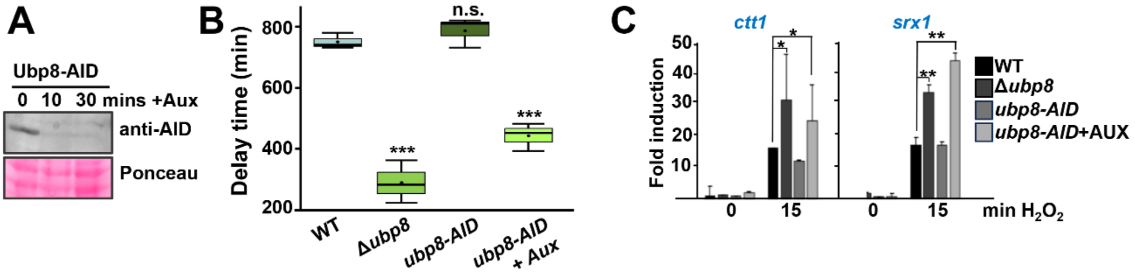

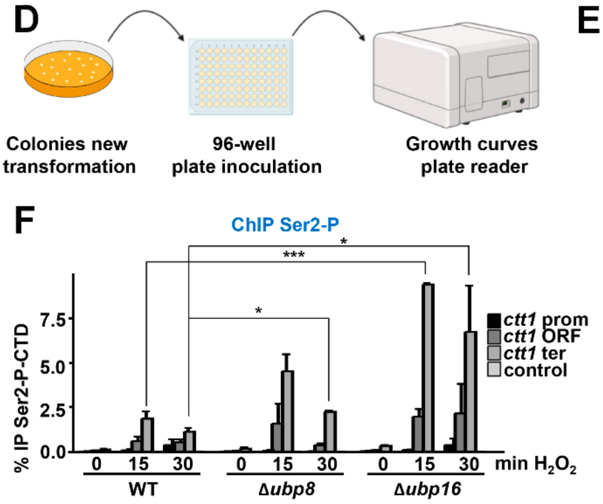

E

Strain		Delay time upon 1 mM H_2O_2 (in min)
972 (WT)		750
RB64 (Δ*ubp8*)		250
New transformation *ubp8::kanMX6*	Clon 1	260
	Clon 2	230
	Clon 3	210
	Clon 4	220
	Clon 5	170
RB65 (Δ*ubp16*)		160
New transformation *ubp16::kanMX6*	Clon 1	130
	Clon 2	100
	Clon 3	90
	Clon 4	120
	Clon 5	150

Fig. S2 Alternative strategies to generate Ubp8 or Ubp16 depleted cells. **A** TCA extracts from cell cultures of strain RB239 (*ubp8-AID*) before and after treatment with 1 mM 5-adamantyl IAA (Aux) at indicated times. Western blot using monoclonal antibody anti-mini-AID is shown. Ponceau was used as loading control. **B** Boxplots representing growth delays, as in Fig. 1D, of 972 (WT), RB64 (Δ*ubp8*), and RB239 (*ubp8-AID*) strains in untreated and treated conditions (1 mM H_2O_2). Cells expressing Ubp8-AID were also untreated (dark green) and pretreated with Aux for 90 min (light green) prior to H_2O_2 addition. ***p<0.001; n.s., non-significative. **C** Relative expression of the stress genes *ctt1* and *srx1* under basal and treated conditions (15 min 1 mM H_2O_2). Ubp8-AID-expressing cells were also pre-treated or untreated with Aux for four hours. Total RNA from 972 (WT), RB64 (Δ*ubp8*), and RB239 (*ubp8-AID*) strains was obtained and quantified by qPCR as in Fig. 1E. *p<0.05; **p<0.01. **D** Graphical scheme for the isolation of new and independent clones of Δ*DUBs* and analysis of H_2O_2 tolerance by growth curves recording. **E** Delay time represented, as in Fig. 1C, from freshly obtained cultures of Δ*ubp8* and Δ*ubp16* isolates following procedure in **D**. **F** Cell cultures from 972 (WT), RB64 (Δ*ubp8*), and RB65 (Δ*ubp16*) strains were treated or not with 1 mM H_2O_2 for 15 and 30 minutes. ChIP experiments using anti-Ser2-P, coupled to qPCR, using same primers as in Fig. 1F. *p<0.05; *** p<0.001.

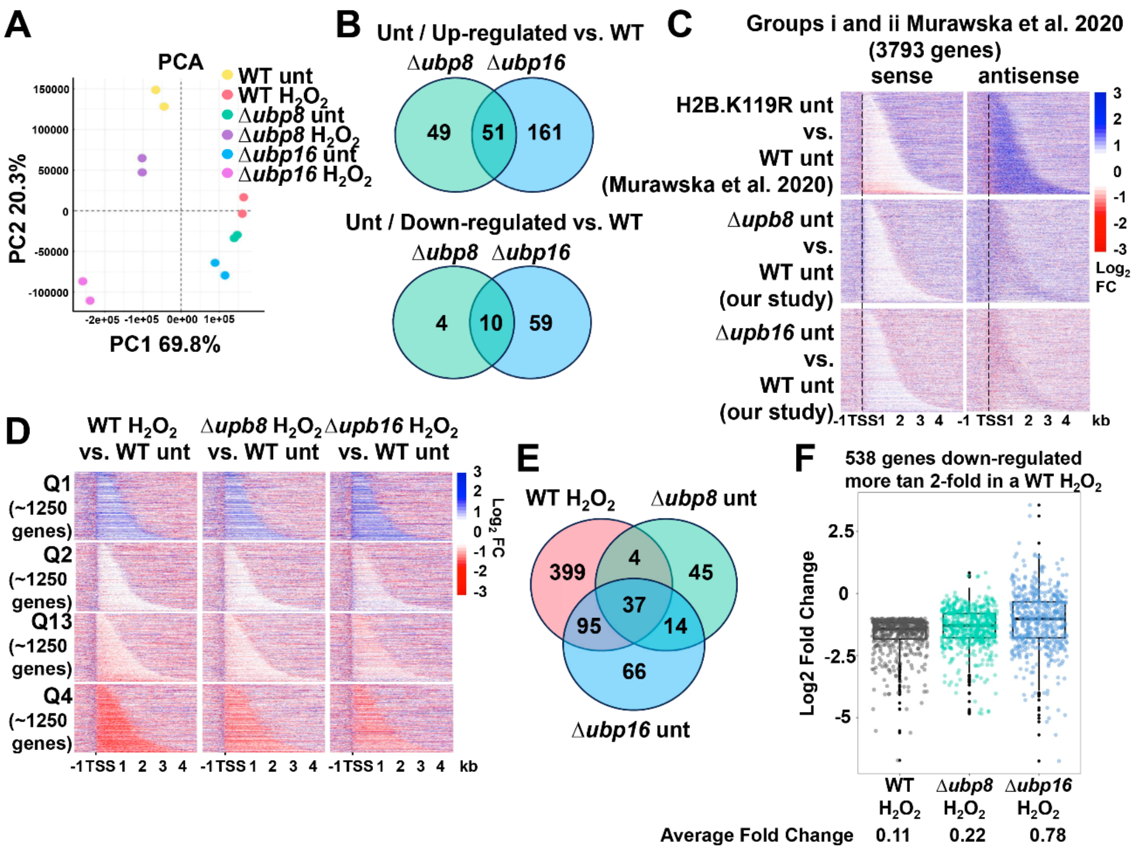

Fig. S3 Transcriptome landscape of DUB mutants showed up-regulated stress response. **A** RNA-seq Principal Component Analysis (PCA) of 972 (WT), RB64 (Δ*ubp8*) and RB65 (Δ*ubp16*) strains treated or not with 1 mM H_2O_2 for 30 minutes. **B** Venn diagrams showing common genes significantly up-regulated (upper diagram) or down-regulated (lower diagrams) in the DUBs mutants in basal conditions. **C** Heatmaps showing Log_2 Fold Changes of normalized coverage reads in 3793 genes selected from [8] in H2B.K119R, and DUBs mutants relative to the WT. Left heatmaps show coverage of coding genes, and right heatmaps show the coverage of antisense of the coding genes. Genes were aligned to the TSS and genomic regions from -1 kb to +5 kb from the TSS are represented. Genes are arranged by length. **D** Heatmaps showing Log_2 Fold Changes of normalized coverage reads of coding genes in the indicated strains relative to WT. Whole genome was divided into four quartiles based on the expression of genes after stress imposition in a WT strain (Q1 more expressed; Q4 less expressed). **E** Venn diagram of common induced genes in the indicated strains and conditions. **F** Box plot representing the expression of the 538 genes down-regulated in a WT after peroxide treatment. Each box represents the Log_2 Fold Change of genes in the indicated strain relative to the WT untreated. The average of the fold changes of the 538 genes in each strain is indicated at the bottom of the panel.

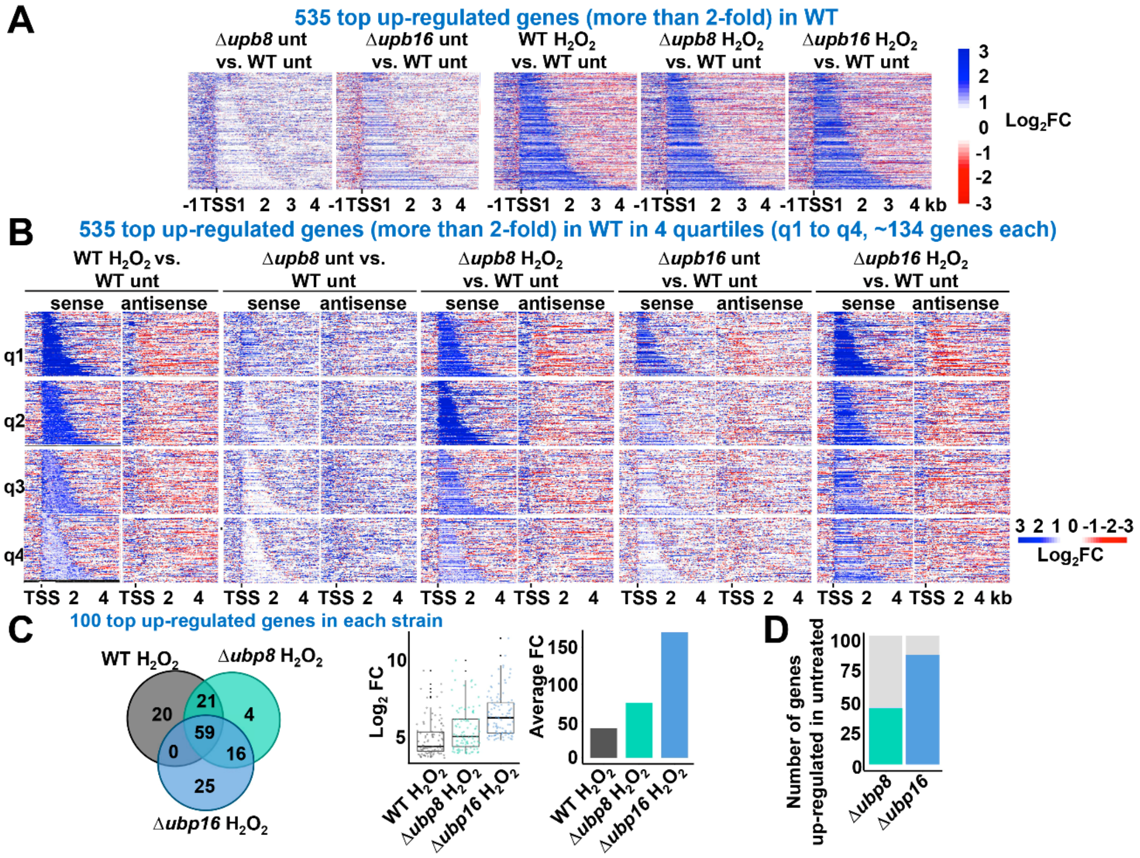

Fig. S4 Transcriptome landscape of DUB mutants showed up-regulated stress response. **A** Heatmaps showing the expression of the up-regulated genes in a WT strain after peroxide treatment. Log_2 Fold Change of normalized coverage is represented for the indicated strains and treatments compared to the WT untreated. Genes were aligned to the TSS and genomic regions from -1 kb to +5 kb from the TSS are represented. Genes are arranged by length. **B** Heatmaps showing expression of coding genes from A divided into quartiles of expression and its antisense expression (q1 more expressed; q4 less expressed). **C** Same as in Fig. 2D for the 100 most up-regulated genes in each strain after peroxide. **D** Same plot as in Fig. 2E with genes from **C**.

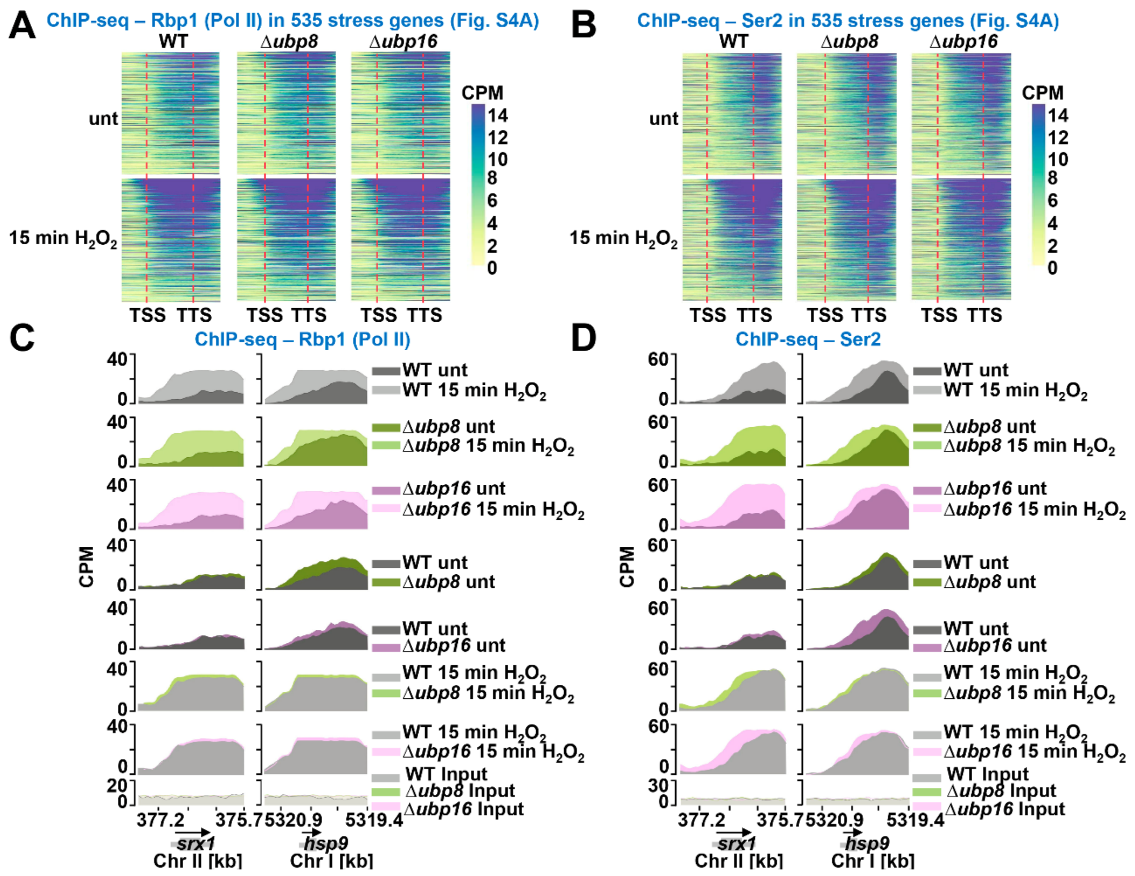

Fig. S5 ChIP-seq analysis of Rbp1-HA (Pol II) and phosphorylated Pol II at Ser2. **A** Heatmaps showing the Rpb1-HA occupancy at the 535 most expressed genes from Fig. S4A. ChIP-seq was performed from strains CS61 (*rpb1-HA*), RB163 (Δ*ubp8 rpb1-HA*) and RB164 (Δ*ubp16 rpb1-HA*), before and after 15 min 1 mM H_2O_2, using anti-HA antibody. Genes bodies were scaled to 1 kb and aligned -500 bp to +500 bp relative to the TSS and TTS respectively. Genes are arranged by expression levels in wild-type cells after H_2O_2. **B** Heatmaps showing phosphorylated Pol II at Ser2 occupancy at the 535 most expressed genes from Fig. S4A. ChIP-seq was performed from strains 972 (WT), RB64 (Δ*ubp8*) and RB65 (Δ*ubp16*) before and after 15 min of 1 mM H_2O_2, using anti-Ser2 antibody. Represented as in **A**. **C** ChIP-seq analysis of Pol II at *srx1* and *hsp9* genes. Line plots represented as in Fig. 2G of strains as in **A**. Different CPM coverage comparisons among strains are overlapped in the different panels, with the following color code: grey for WT, green for Δ*ubp8* and pink for Δ*ubp16*. **D** ChIP-seq analysis of Pol II phosphorylated at Ser2 at *srx1* and *hsp9* genes. Line plots and panels represented as in **C** of strains as in **B**.

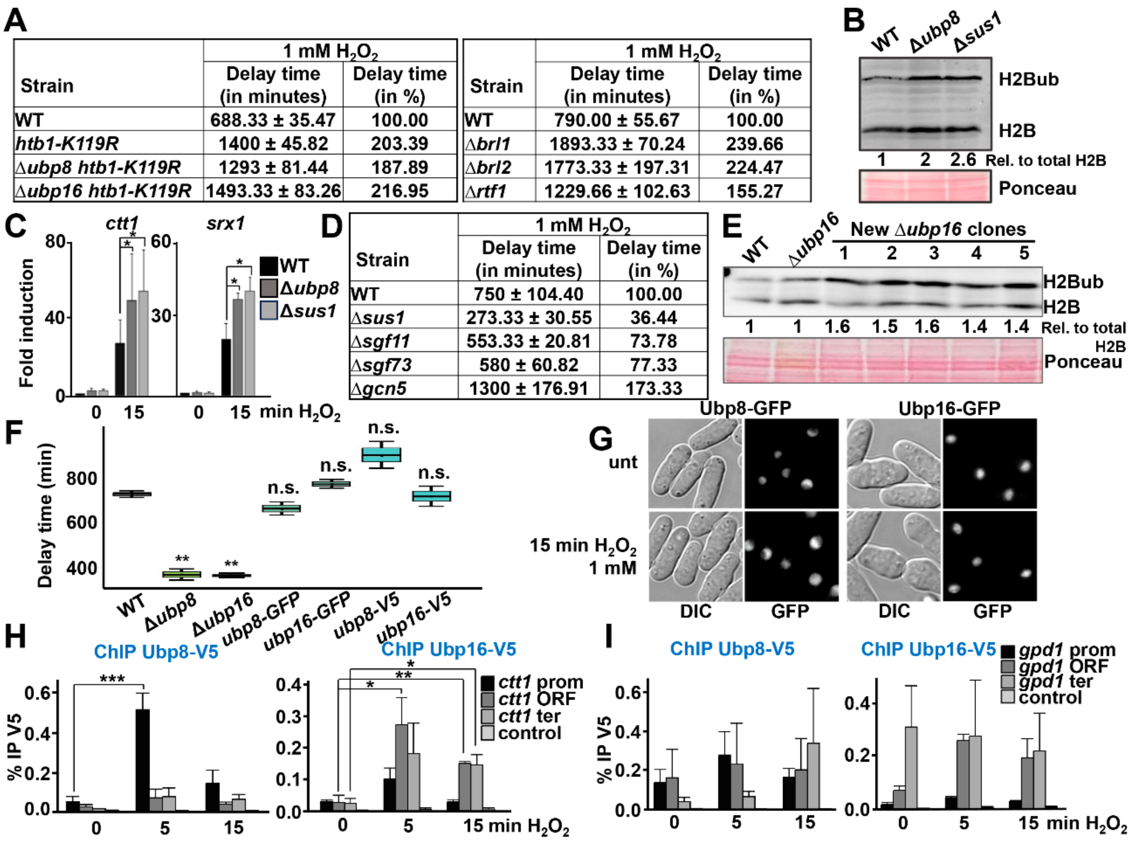

Strain	1 mM H_2O_2 Delay time (in minutes)	Delay time (in %)
WT	688.33 ± 35.47	100.00
htb1-K119R	1400 ± 45.82	203.39
Δ*ubp8 htb1-K119R*	1293 ± 81.44	187.89
Δ*ubp16 htb1-K119R*	1493.33 ± 83.26	216.95

Strain	1 mM H_2O_2 Delay time (in minutes)	Delay time (in %)
WT	790.00 ± 55.67	100.00
Δ*brl1*	1893.33 ± 70.24	239.66
Δ*brl2*	1773.33 ± 197.31	224.47
Δ*rtf1*	1229.66 ± 102.63	155.27

Strain	1 mM H_2O_2 Delay time (in minutes)	Delay time (in %)
WT	750 ± 104.40	100.00
Δ*sus1*	273.33 ± 30.55	36.44
Δ*sgf11*	553.33 ± 20.81	73.78
Δ*sgf73*	580 ± 60.82	77.33
Δ*gcn5*	1300 ± 176.91	173.33

Fig. S6 Mutations of H2B ubiquitination regulators affect tolerance to oxidative stress. **A** Delay time calculated from Fig. 3B growth curves, represented as in Fig. 1C. **B** TCA extracts from 972 (WT), RB64 (Δ*ubp8*), and RB166 (Δ*sus1*) strains were obtained in logarithmic phase. Western blot using anti-H2B antibody is shown as in Fig. 4B. Quantification of H2Bub was performed as described in Fig. 3D. **C** Relative expression of the stress genes *ctt1* and *srx1* under basal and treated conditions (15 min 1 mM H_2O_2). Cell cultures from strains as in **B** and RNA levels were determined by qPCR as in Fig. 1E. **D** Delay time calculated from Fig. 3C growth curves, represented as described in Fig. 1C. **E** TCA extracts from Δ*ubp16* transformants from Fig. S2E were obtained, and western blot was performed as in Fig. 4B. Quantification of H2Bub was performed as described in Fig. 3D. **F** Boxplots representing delay times, as in Fig. 1D, of strains 972 (WT), RB64 (Δ*ubp8*), RB65 (Δ*ubp16*), RB171 (*ubp8-GFP*), RB172 (*ubp16-GFP*), RB181 (*ubp8-V5*) and RB180 (*ubp16-V5*). $^{**}p<0.01$; n.s., non-significative. **G** Fluorescence microscopy showing localization of Ubp8 and Ubp16 in the nucleus. Cell cultures from strains RB171 (*ubp8-GFP*) and RB172 (*ubp16-GFP*) were treated or not with 1 mM H_2O_2. **H** Both DUBs are recruited to the *ctt1* gene upon H_2O_2 treatment. Cell cultures of strains RB181 (*ubp8-V5*) and RB180 (*ubp16-V5*), treated or not with 1 mM H_2O_2 at indicated times. ChIP experiments using anti-V5 antibody, coupled to qPCR, were performed using same primers as in Fig. 1F. $^{*}p<0.05$; $^{**}p<0.01$; $^{***}p<0.001$. **I** Both DUBs are recruited to the *gpd1* gene upon H_2O_2 treatment. ChIP experiments using strains and conditions as in **H**. qPCR was performed using primers amplifying for promoter, ORF, and terminator regions of *gpd1* gene. Each column represents the mean value and SD, calculated from three biological replicates.

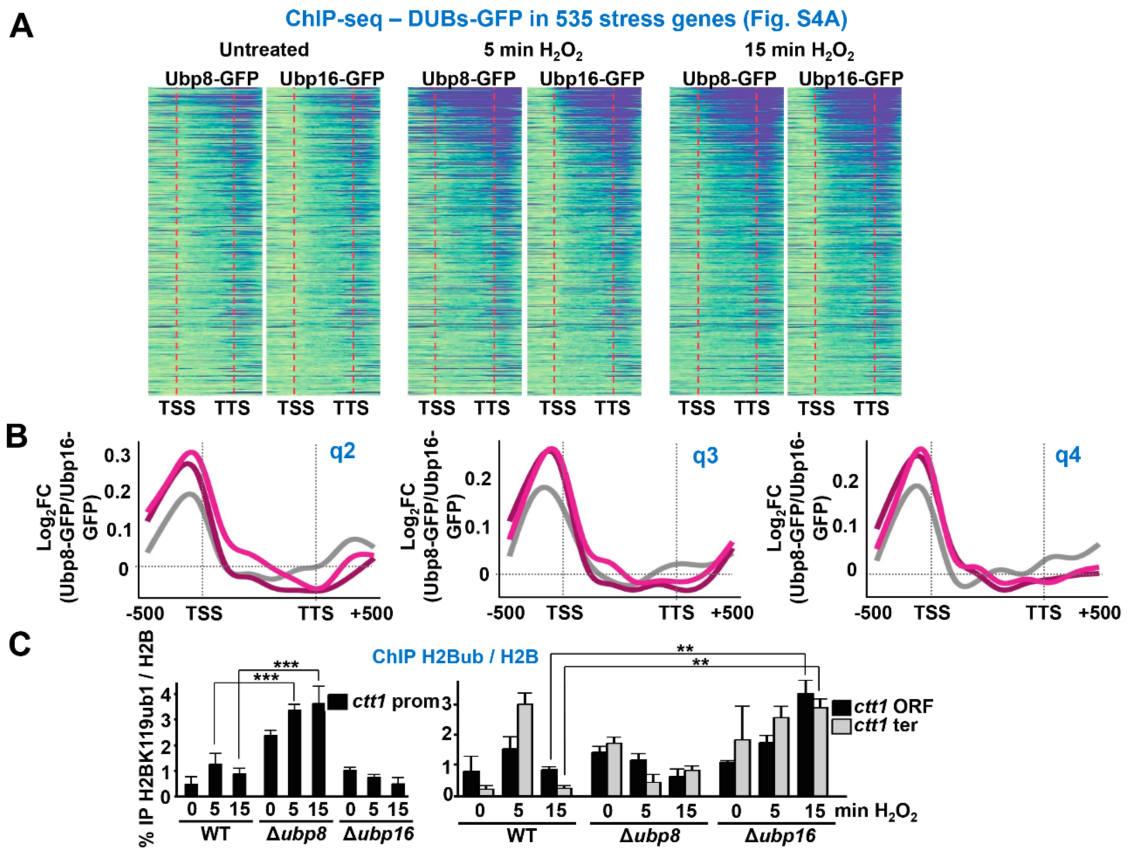

Fig. S7 ChIP-seq analysis of Ubp8 and Ubp16. **A** Heatmaps showing DUBs occupancy at the 535 most expressed genes from Fig. S4A. ChIP-seq was performed from strains RB171 (Ubp8-GFP) and RB172 (Ubp16-GFP) before and after 5 and 15 min of 1 mM H_2O_2. Represented as in Fig. S5A. **B** Line plot representing Log_2 Fold Changes of Ubp8-GFP coverage relative to Ubp16-GFP. Average CPM coverage from genes arranged in quartiles of expression from Fig. S4B (q1, first quartile, is shown in Fig. 3G). Line plots are represented as in Fig. 2H. **C** H2Bub is accumulated at *ctt1* gene in Δ*ubp8* and Δ*ubp16* strains. Cell cultures of strains 972 (WT), RB64 (Δ*ubp8*), and RB65 (Δ*ubp16*) were treated or not with 1 mM H_2O_2 at indicated times. ChIP experiments were performed using antibodies anti-H2B-K119ub1 or anti-H2B, coupled to qPCR, using same primers as in Fig. 1F. The percentage of immunoprecipitated H2Bub versus total H2B is represented. $^{**}p<0.01$; $^{***}p<0.001$.

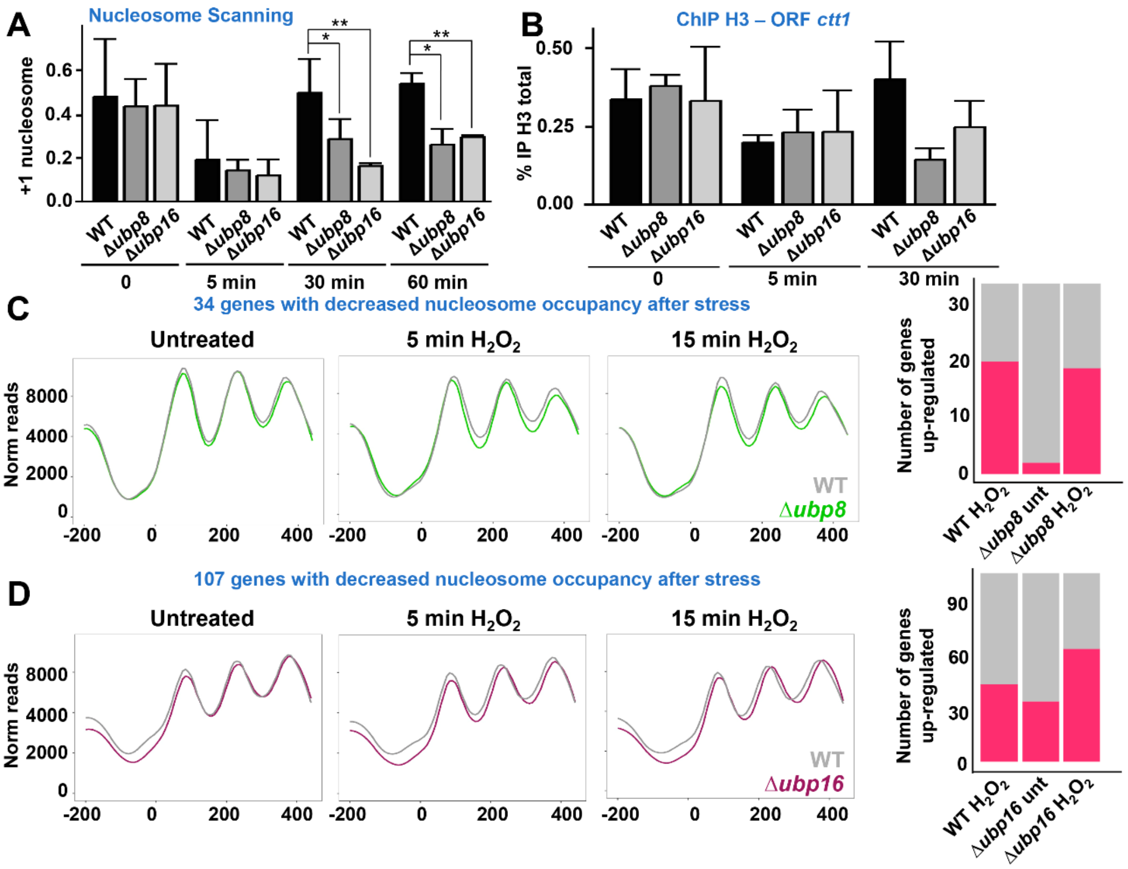

Fig. S8 Nucleosome eviction upon H_2O_2 treatment is more sustained in cells lacking DUBs. **A** Bar plot representing the *ctt1* +1 nucleosome positioning values (peaks) obtained from Fig. 5A. Each column represents the mean value and SD, calculated from three biological replicates. *p<0.05; **p<0.01. **B** ChIP experiments from 972 (WT), RB64 (Δ*ubp8*), and RB65 (Δ*ubp16*) strains at before and after 5 or 30 min of 1 mM H_2O_2, using anti-H3 antibody coupled to qPCR using primers amplifying the ORF region of the *ctt1* gene. Each column represents the mean value and SD, calculated from three biological replicates. **C,D** Several genes are highly sensitive to increased H2Bub showing a decreased nucleosome occupancy. **C** Line plots represent average normalized reads of 34 genes which are exhibiting decreased nucleosome occupancy after 5 minutes of H_2O_2 treatment in Δ*ubp8* strain compared to wild-type values (y-axis). Genes were aligned to the TSS (0) and -200 to +500 bp regions are represented (x-axis) for untreated conditions (left panel), 5 minutes (central panel), and 15 minutes (right panel) after 1 mM H_2O_2 treatment. Each line represents one strain: WT in gray, Δ*ubp8* in green. Bar plots represent the number of stress-induced genes among these 34 genes at indicated strains and treatments. **D** Average reads of 107 genes showing decreased nucleosome occupancy after 5 minutes of H_2O_2 treatment in Δ*ubp16* strain. Results are represented as in **C**. Lines represents one strain: WT in gray, Δ*ubp16* in magenta. Bar plot represent the number of stress-induced genes among these 107 genes at the indicated strains and treatments.

A

Strain	1 mM H_2O_2	
	Delay time (in minutes)	Delay time (in %)
WT	783.33 ± 41.63	100.00
Δ*ubp8*	443.33 ± 35.11	55.88
Δ*ubp16*	376.66 ± 15.27	47.47
Δ*hrp3*	1743.66 ± 110.15	219.74
Δ*snf22*	1680 ± 95.39	211.76
Δ*ubp8* Δ*snf22*	926.66 ± 32.14	116.80
Δ*ubp8* Δ*hrp3*	740 ± 55.67	93.27
Δ*ubp16* Δ*snf22*	943.33 ± 68.06	118.90
Δ*ubp16* Δ*hrp3*	676.66 ± 32.14	85.29

B

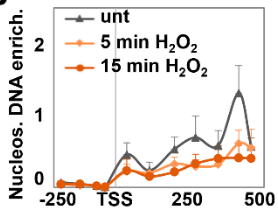

C

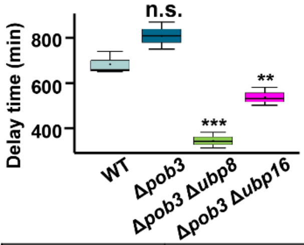

Strain	1 mM H_2O_2	
	Delay time (in minutes)	Delay time (in %)
WT	683.33 ± 49.33	100.00
Δ*pob3*	810 ± 60	118.54
Δ*ubp8* Δ*pob3*	343.33 ± 35.11	50.24
Δ*ubp16* Δ*pob3*	536.66 ± 40.41	78.54

D

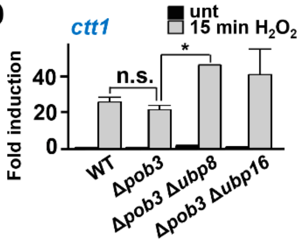

E

Strain	1 mM H_2O_2	
	Delay time (in minutes)	Delay time (in %)
WT	678 ± 120.23	100.00
Δ*ubp8*	264 ± 65.03	34.47
Δ*ubp16*	183 ± 95.33	27.10
Δ*set1*	1317.17 ± 173.5	194.02
Δ*ubp8* Δ*set1*	536.75 ± 123.28	83.04
Δ*ubp16* Δ*set1*	1008.13 ± 210.37	148.5
hht2-K4R	906.25 ± 159.63	133.49
Δ*ubp8 hht2-K4R*	743.33 ± 121.93	60.39
Δ*ubp16 hht2-K4R*	410 ± 69.89	109.5

Strain	1 mM H_2O_2	
	Delay time (in minutes)	Delay time (in %)
WT	678 ± 120.23	100.00
Δ*ubp8*	264 ± 65.03	34.47
Δ*ubp16*	183 ± 95.33	27.10
Δ*gcn5*	1162.5 ± 128.03	171.24
Δ*ubp8* Δ*gcn5*	318.57 ± 129.92	46.93
Δ*ubp16* Δ*gcn5*	1155.05 ± 177.02	170.14
Δ*ada2*	862.5 ± 139.61	127.05
Δ*ubp8* Δ*ada2*	220 ± 21.21	32.41
Δ*ubp16* Δ*ada2*	638 ± 102.55	93.98

Fig. S9 Genetic interactions of H2Bub with chromatin remodelers, FACT, SAGA or COMPASS. **A** Delay time calculated from Fig. 6C, represented as described in Fig. 1C. **B** Nucleosome scanning of the *ctt1* gene before (triangles) and after (5 min, diamonds; 15 min, circles) 1 mM H_2O_2 stress imposition was performed in RB184 (Δ*pob3*) as in Fig. 5A (left panel). **C** Boxplots representing delay times of strains as in **B**; performed as in Fig. 1CD. **p<0.01; ***p<0.005; n.s., non-significative. **D** Relative expression of *ctt1* in strains 972 (WT), RB184 (Δ*pob3*), RB185 (Δ*ubp8* Δ*pob3*), and RB186 (Δ*ubp16* Δ*pob3*), before and after 1 mM H_2O_2, was quantified as described in Fig. 1E. *p<0.05; **p<0.01. **E** Delay time calculated from Fig. 7A and 7D, represented as described in Fig. 1C.

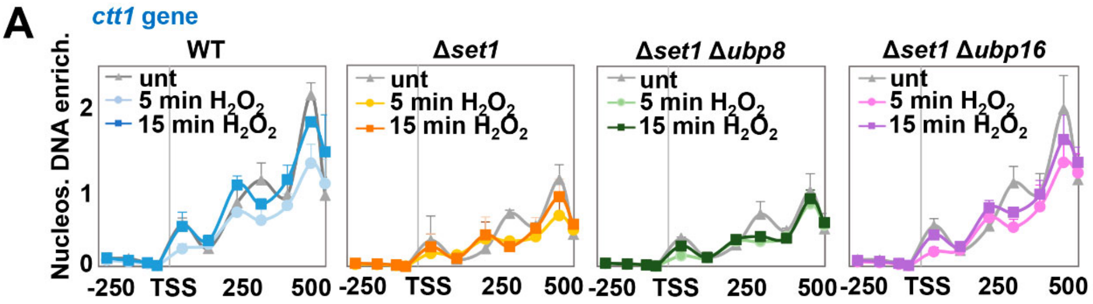

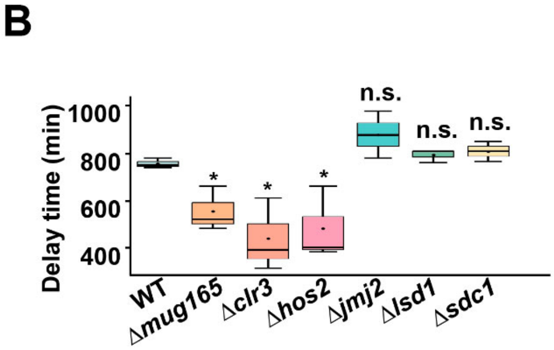

Strain	1 mM H_2O_2	
	Delay time (in minutes)	Delay time (in %)
WT	756.66 ± 20.81	100.00
Δ*mug165*	553.33 ± 94.51	73.13
Δ*clr3*	436.66 ± 155.34	57.71
Δ*hos2*	480 ± 156.20	63.44
Δ*jmj2*	880 ± 100	116.30
Δ*lsd1*	793.33 ± 28.86	104.85
Δ*sdc1*	808.33 ± 42.52	106.83

Fig. S10 Chromatin remodelers and histone marks writers genetically interact with DUBs function. **A** Nucleosome scanning of the *ctt1* was performed using strains 972, JE28 (Δ*set1*), RB118 (Δ*ubp8* Δ*set1*) and RB119 (Δ*ubp16* Δ*set1*), represented as described in Fig. 5A. **B** Boxplots representing delay times of strains 972 (WT), PG105 (Δ*mug165*), PG112 (Δ*clr3*), PG113 (Δ*hos2*), RB153 (Δ*jmj2*), RB218 (Δ*lsd1*), and RB219 (Δ*sdc1*) (left panel). **p*<0.05; n.s., non-significative. Respective delay times are calculated and represented as in Fig. 1C (right panel).
