# Supplementary material for: Distinct roles of histone H2B ubiquitination at promoters and coding regions of Pol II-transcribed stress genes
Source: Genome Biol. 2025 Dec 9;26:419. doi: 10.1186/s13059-025-03891-1 (PMC12687531; doi:10.1186/s13059-025-03891-1)
Supplement: Supplementary file 2 — Additional file 2: Table S1. Strains used in this study. [file 13059_2025_3891_MOESM2_ESM.pdf]

ADDITIONAL FILE 2

Table S1. Strains used in this study

Strain	Genotype	Origin
972	h^{-}	[49]
RB64	h^{-} *ubp8::natMX6*	This work
RB65	h^{-} *ubp16::natMX6*	This work
CS61	h^{-} *rpb1.rpb1-HA::kanMX6*	This work
RB163	h^{-} *ubp8::natMX6 rpb1.rpb1-HA::kanMX6*	This work
RB164	h^{-} *ubp16::natMX6 rpb1.rpb1-HA::kanMX6*	This work
RB203	$h^{?}$ *htb1-K119R::kanMX6*	This work
RB100	h^{-} *ubp8::natMX6 htb1-K119R-FLAG::kanMX6*	This work
RB101	h^{-} *ubp16::natMX6 htb1-K119R-FLAG::kanMX6*	This work
RB158	$h^{?}$ *brl1::hphMX6*	This work
RB204	$h^{?}$ *brl2::hphMX6*	This work
RB191	$h^{?}$ *rtf1::hphMX6*	This work
RB166	$h^{?}$ *sus1::hphMX6*	This work
RB187	$h^{?}$ *sgf11::hphMX6*	This work
RB192	$h^{?}$ *sgf73::hphMX6*	This work
KGY15253	h^{-} *htb1-FLAG::kanMX6 ade6-M21X*	[33]
KGY15388	h^{-} *htb1-K119R-FLAG::kanMX6 ade6-M21X*	[33]
RB88	h^{-} *ubp8::natMX6 htb1-FLAG::kanMX6 ade-M21X*	This work
RB89	h^{-} *ubp16::natMX6 htb1-FLAG::kanMX6 ade-M21X*	This work
RB117	h^{-} *ubp8::hphMX6 ubp16::natMX6 htb1-FLAG::kanMX6 ade-M21X*	This work
RB181	$h^{?}$ *ubp8-V5::natMX6*	This work
RB180	$h^{?}$ *ubp16-V5::natMX6*	This work
JE28	h^{-} *set1::kanMX6*	[40]
FY6078	h^{-} *h3.2K9RK14R h3.1/h4.1::his3+ h3.3/h4.3::arg3+ otr1R(SphI):ade6+*	[50]
MS112	h^{+} *gcn5::kanMX6*	[24]
JT561	h^{-} *h3.2K4R h3.1/h4.1::his3+ h3.3/h4.3::arg3+ otr1R(SphI)::ade6+*	[51]
RB211	$h^{?}$ *spf1-TAP::kanMX6*	This work
RB214	$h^{?}$ *spf1-TAP::kanMX6 ubp8::natMX6*	This work
RB215	$h^{?}$ *spf1-TAP::kanMX6 ubp16::natMX6*	This work
MP8	h^{+} *gcn5-HA::ura4+*	This work
RB216	h^{+} *ubp16::natMX6 gcn5-HA::ura4+*	This work

RB217	h^{+} *ubp16::natMX6 gcn5-HA::ura4+*	This work
JA2777	$h^{?}$ *snf22-myc::kanMX6*	[36]
RB195	$h^{?}$ *snf22-myc::kanMX6 ubp8::natMX6*	This work
RB196	$h^{?}$ *snf22-myc::kanMX6 ubp16::natMX6*	This work
RB244	$h^{?}$ *snf22-myc::kanMX6 htb1-K119R:hphMX6*	This work
JA2653	$h^{?}$ *hrp3-myc::kanMX6*	[36]
RB193	$h^{?}$ *hrp3-myc::kanMX6 ubp8::natMX6*	This work
RB194	$h^{?}$ *hrp3-myc::kanMX6 ubp16::natMX6*	This work
RB243	$h^{?}$ *hrp3-myc::kanMX6 htb1-K119R:hphMX6*	This work
IV84	h^{+} *snf22::natMX6*	[36]
IV83	h^{+} *hrp3::natMX6*	[36]
RB199	$h^{?}$ *snf22::natMX6 ubp8::kanMX6*	This work
RB200	$h^{?}$ *snf22::natMX6 ubp16::kanMX6*	This work
RB201	$h^{?}$ *hrp3::natMX6 ubp8::kanMX6*	This work
RB202	$h^{?}$ *hrp3::natMX6 ubp16::kanMX6*	This work
RB184	$h^{?}$ *pob3::kanMX6*	This work
RB185	$h^{?}$ *pob3::kanMX6 ubp8::natMX6*	This work
RB186	$h^{?}$ *pob3::kanMX6 ubp16::natMX6*	This work
RB156	h^{-} *ubp8::natMX6 h3.2K4R h3.1/h4.1::his3+ h3.3/h4.3::arg3+ otr1R(SphI):ade6+*	This work
RB157	h^{-} *ubp16::natMX6 h3.2K4R h3.1/h4.1::his3+ h3.3/h4.3::arg3+ otr1R(SphI):ade6+*	This work
RB118	h^{-} *set1::kanMX6 ubp8::natMX6*	This work
RB119	h^{-} *set1::kanMX6 ubp16::natMX6*	This work
RB165	h^{+} *gcn5::kanMX6 ubp8::natMX6*	This work
RB168	h^{+} *gcn5::kanMX6 ubp16::natMX6*	This work
IV70	$h^{?}$ *ada2::kanMX6*	This work
RB177	$h^{?}$ *ada2::kanMX6 ubp8::natMX6*	This work
RB178	$h^{?}$ *ada2::kanMX6 ubp16::natMX6*	This work
AZ74	h^{-} *pka1::kanMX6*	[52]
AV18	h^{-} *sty1::kanMX6*	[53]
AZ63	h^{-} *pyp1::kanMX6*	This work
MS98	h^{-} *atf1::natMX6*	[54]
SB442	$h^{?}$ *ubp8::kanMX6 atf1::natMX6*	This work
SB443	$h^{?}$ *ubp16::kanMX6 atf1::natMX6*	This work
RB239	h^{-} *adh1p-OsTir1-F74A-adh1ter::ura4 ura4-D18 ubp8-AID::kanMX6*	This work
RB171	$h^{?}$ *ubp8-GFP::kanMX6*	This work

RB172	*h*$^{?}$ *ubp16-GFP::kanMX6*	This work
PG105	*h*$^{?}$ *mug165::kanMX6*	This work
PG112	*h*$^{+}$ *clr3::kanMX6*	This work
PG113	*h*$^{+}$ *hos2::kanMX6*	This work
RB153	*h*$^{?}$ *jmj2::kanMX6*	This work
RB218	*h*$^{?}$ *lsd1::kanMX6*	This work
RB219	*h*$^{?}$ *sdc1::kanMX6*	This work
